# Progression of swine fecal microbiota during early stages of life and its association with performance: a longitudinal study

**DOI:** 10.1186/s12866-024-03336-y

**Published:** 2024-05-25

**Authors:** Maddison Arsenault, Brandon Lillie, Khurram Nadeem, Ehsan Khafipour, Abdolvahab Farzan

**Affiliations:** 1 Department of Pathobiology, University of Guelph, Guelph, ON Canada; 2 Department of Mathematics & Statistics, University of Guelph, Guelph, ON Canada; 3 Cargill Animal Nutrition, Minneapolis, MN USA; 4 Department of Population Medicine, University of Guelph, Guelph, ON Canada

**Keywords:** Swine, Microbiome progression, Longitudinal study, Productivity

## Abstract

**Background:**

It is vital to understand healthy gut microbiota composition throughout early life stages when environments are changing, and immunity is developing. There are limited large-scale longitudinal studies classifying healthy succession of swine microbiota. The objectives of this study were to (a) determine the microbiota composition of fecal samples collected from piglets within a few days after birth until one-week post-weaning, and (b) investigate the associations of early fecal microbiota with pig growth performance in nursery and later growing stages. Fecal samples were collected from nine cohorts of 40 pigs (*n* = 360) from distinct farrowing sources in Ontario and Quebec, Canada at four timepoints from birth to one-week post-weaning, with pig body weight was recorded at each fecal sampling.

**Results:**

Microbiota was dominated by the phyla Firmicutes, Bacteroides and Proteobacteria. There were notable differences in genera abundance between pigs from different provinces and farming systems. Over the early life stage, the genera *Bacteroides*, *Escherichia/Shigella*, and *Clostridium* cluster *XIVa* were abundant preweaning, while *Prevotella* dominated post-weaning. Hierarchical clustering identified three major stages of microbiota development, each associated with distinct composition. Stage one occurs from birth to 7 days, stage two from 7 days after birth until weaning, and stage three from weaning to one-week post-weaning. Three enterotypes were identified in stage two that showed differences in growth before weaning, and in the grower production stage. Piglets with a microbiota enterotype characterized by higher abundance of *Prevotella* and unclassified *Ruminococcaceae* had lower growth performance in the pre-weaning stage, and the growing stage.

**Conclusion:**

These findings help identify the timing of microbiota shifts across early swine life which may be the optimal time for external intervention to shift the microbiota to a beneficial state. The project findings should help decrease antimicrobial use, increase animal welfare, and have positive economic impacts.

**Supplementary Information:**

The online version contains supplementary material available at 10.1186/s12866-024-03336-y.

## Background

The swine gut microbiota is composed of many microbes that occupy ecological niches within the gastrointestinal (GI) tract [1]. This microbial community interacts with the host pig to provide immunological and metabolic benefits [2–4], and it is an area of focus for interventions to beneficially alter microbiota to improve swine health and productivity [5, 6]. With implementation of restrictions on antimicrobial use, animal agricultural sectors must transitioning away from subtherapeutic use of antimicrobials to alternatives like prebiotics, probiotics, postbiotics and phytogenics to alter gut microbiota in favour of beneficial bacteria [7, 8].

The pig gut microbiota is dynamic and evolves from birth across all production stages until a stable microbiome community is reached in the grow/finish production stage [9]. Fecal microbiota is an important area of study as it is a good indicator of which microbes are present in a pig’s gut [10]. It has been previously demonstrated that the early fecal microbiota in piglets can impact later microbiota composition and swine productivity into adulthood [11]. Environmental factors dictate a pig’s early microbial exposure, thus which microbes colonize the gastrointestinal tract. Microbiota also varies among individual pigs and can be influenced by housing and management system, diet, antimicrobial use, and/or other environmental factors, all of which vary considerably across the first month of a pig’s life [12]. At farrowing, microorganisms are vertically transferred from the sow to piglet to prime the GI tract for further colonization by environmental microbes [13, 14]. During the suckling phase, the piglet gut microbiota contains high abundance of *Lactobacillus* and *Escherichia/Shigella* spp. bacteria [15, 16]. At this production stage, the immune system is in development so there are high levels of potentially pathogenic bacteria from the genera *Escherichia* and *Clostridium*, but maternal IgA antibodies pass through the milk to strengthen piglet’s immune system and defend against potential pathogens [17, 18]. It is valuable to understand the gut microbiota in suckling piglets as it is predictive of occurrence and severity of post-weaning diarrhea, and growth performance of adult pigs [19, 20].

In controlled studies, it is easy to examine the impact of singular environmental factors on the gut microbiota. However, under commercial production systems, several factors may interact and their associations with microbiota are difficult to elucidate. Therefore, observational studies are needed to explore the impact of multiple factors such as age, antibiotic use, and other housing conditions on pig gut microbiota. This will help to understand the association of early-stage microbiota in performance across commercial farms and to develop effective strategies in alignment with commercial farming practices. Fecal microbiota has high similarity to the microbiota of large intestines and sample collection is easily obtained without animal sacrifice [10]. The objectives of this study were to (a) determine the microbiota composition of fecal samples collected from piglets within a few days after birth until one-week post-weaning, and (b) investigate associations between early fecal microbiota and pig growth performance in pre-weaning and growing stages.

## Methods

### Animals and sample collection

Animal use in this project was approved under the University of Guelph Animal Care Committee (AUP #4122) and follows Canadian Council of Animal Care guidelines.

Nine cohorts of 40 pigs, for a total of 360 pigs were included in this study. Cohorts were selected from four swine farms located in Ontario (ON) and five swine farms in Quebec (QC). Furthermore, five cohorts were raised under conventional farming system management (CONV; 3 ON, 2 QC), and four cohorts were raised without antibiotics (RWA; 1 ON, 3 QC) (Table 1). For each cohort 40 piglets were selected from 10 litters [4 piglets per litter]. Sow parity and litter size were -recorded. Each of the nine farms included in this study completed a comprehensive survey detailing rearing practices. This survey included details on the size of the farm (number of pigs and size of barn), production strategies, biosecurity protocols, feed type and ingredients, medication, vaccine and antibiotic administration, disease outbreaks, and swine mortality (Table S1, Additional File 1).

**Table 1:** Number of samples (collected; analyzed), parity of the sows (primiparous, P; multiparous, M), nursery location (on-site, On; off-site, Off), and the median sampling age and body weight (BW) of pigs and their standard deviation (SD) at each sampling timepoint (TP). Pigs are grouped all together (overall), by system (conventional, CONV; raised without antibiotics, RWA), province (Ontario, ON; Quebec, QC), and by cohort (cohort 1 – 9)

Group	Province	System	Parity		Nurs-ery site	Age at Sampling (d)				BW (kg)				Diarrhea Occurrences			
			***P***	***M***		***Median (SD)***				***Median (SD)***							
						TP1	TP2	TP3	TP4	TP1	TP2	TP3	TP4	TP1	TP2	TP3	TP4
**Overall**	**-**	**-**	**35**	**55**		**4 (1) **	**11 (2)**	**18 (2)**	27 (3)	2.1 (0.7)	3.9 (1.2)	6 (2.4)	7.2 (2.7)	-	-	-	-
**CONV**	**-**	**-**	23	27		4 (1)	11 (1)	18 (3)	27 (3)	2.1 (0.8)	3.8 (1.2)	5.9 (2.5)	7.3 (2.8)	3	0	7	7
**RWA**	**-**	**-**	12	28		4 (1)	11 (2)	18 (2)	26 (4)	2.1 (0.8)	4.2 (1.1)	6.2 (2.5)	6.9 (2.5)	9	12	16	22
**Ontario**	**-**	**-**	12	24		4 (1)	11 (3)	18 (1)	25 (3)	1.8 (0.8)	3.7 (1.3)	5.3 (1.6)	6.1 (2.1)	-	-	-	-
**Quebec**	**-**	**-**	19	31		4 (1)	11 (1)	20 (8)	28 (7)	2.3 (0.5)	4.1 (1)	7 (2.4)	7.9 (2.8)	12	12	23	29
**Cohort 1**	ON	CONV	6	4	On	3 (0)	10 (0)	18 (0)	27 (0)	1.7 (0.6)	3.2 (1.3)	4.9 (1.7)	5.7 (2.3)	-	-	-	-
**Cohort 2**	ON	CONV	1	9	On	5 (2)	14 (2)	18 (2)	24 (2)	1.7 (0.5)	4.2 (0.6)	5.3 (1.6)	5.7 (1.9)	-	-	-	-
**Cohort 3**	ON	CONV	8	2	NA	3 (0)	11 (0)	17 (0)	25 (0)	1.6 (0.6)	3.6 (1.2)	5.1 (1.7)	6 (1.4)	-	-	-	-
**Cohort 4**	ON	RWA	4	6	Off	4 (0)	10.5 (0)	16.5 (0)	25.5 (0)	2.3 (0.4)	3.8 (0.8)	5.6 (1.3)	7.8 (1.5)	-	-	-	-
**Cohort 5**	QC	RWA	4	6	On	1.5 (0)	11.5 (1)	18.5 (1)	26.5 (1)	2.2 (0.7)	4.2 (1.3)	6.2 (1.7)	6.6 (1.9)	0	0	0	14
**Cohort 6**	QC	RWA	3	7	Off	4 (1)	11 (1)	18 (1)	25 (1)	2.3 (0.9)	4.3 (1.7)	5.9 (2.5)	6.8 (1.9)	0	2	0	7
**Cohort 7**	QC	RWA	4	6	On	4 (0)	11 (0)	27 (0)	34 (0)	2.3 (0.6)	3.9 (0.9)	8.5 (1.3)	9.5 (1.4)	9	10	16	1
**Cohort 8**	QC	CONV	4	6	On	4 (1)	NA (1)	19 (1)	27 (1)	2.2 (0.6)	3.9 (0.8)	6.5 (1.4)	7.6 (1.9)	2	0	2	0
**Cohort 9**	QC	CONV			On	4 (1)	11 (NA)	25 (1)	33 (1)	2.3 (0.5)	4.3 (0.9)	8.3 (1.2)	10 (1.4)	1	0	5	7

Fecal swabs (BD BBL single swab sterile, cat # 220,115, Fisher Scientific) were obtained from each piglet at four timepoints including timepoint 1 (TP1; mean ± standard deviation = 4 ± 1 d after birth), timepoint 2 (TP2; 11 ± 1 d after birth), timepoint 3 (TP3; 1 d prior to weaning), and timepoint 4 (TP4; 1 wk post-weaning) (Table 2). The swabs were collected by inserting the swab into the rectum and rotating to collect feces. The samples were transferred on ice to lab and stored in -80 °C until DNA extraction. In Quebec pigs, if there was visual evidence of recent piglet diarrhea at each sampling timepoint, the occurrence of diarrhea was recorded (Table 2). Diarrheal occurrences in Ontario pigs were not recorded or shared with the research team.

**Table 2:** Mean age, mortality rates, and diarrhea occurrences of pigs at four sample collection times across all nine cohorts in the study

Sampling Timepoint	Associated Production Stage or Transition	Mean Age (± SD) (d)	Mortality Rate	Number of Diarrhea Occurrences^a^	Number of Pigs Sampled
**TP1**	Farrowing	3.71 (1.12)	0.00%	12	360
**TP2**	Suckling	10.01 (3.73)	2.78%	12	350
**TP3**	Weaning	18.97 (4.58)	0.55%	23	348
**TP4**	Nursery	26.68 (5.61)	0.28%	29	347
		**Total**	3.61%	65	1405

^*a*^*Diarrhea occurrence was defined as a pig exhibiting diarrhea symptoms at the time of sampling and only recorded in Quebec cohorts*

Pigs were weighed at each of the four fecal sampling timepoints, and heart girth (HG) was measured two times for each pig; at the end of the nursery stage (TP5; mean ± standard deviation = 60 ± 7 d after birth), and once during the growing stage (TP6; 97 ± 15 d) (Table 3)**.** Heart girth was used as a metric of productivity for pigs past the nursery stage as it is not feasible to obtain accurate body weight (BW) measurements due to their size. Pre-weaning ADG (PW-ADG) was calculated as the difference in BW (g) from timepoint one to timepoint three divided by the difference in pig age between the timepoints (Table 3). Growing stage HG daily gain (G-HGDG) from TP5 to TP6 was calculated to examine the growth performance of pigs later in life (Table 3). The G-HGDG was calculated as the difference in HG (in) from end of nursery to middle of growing stage divided by the difference in pig age between the two times.

**Table 3:** Population performance metrics [median (SD)] including pre-weaning average daily gain (ADG) from sampling timepoint 1 to sampling timepoint 3, and heart girth daily gain (HGDG) of pigs in the grower stage (G-HGDG). Additionally, the age of pigs at timepoint (TP) 5 and 6 which denote the period over HGDG was calculated. Overall, by system (conventional, CONV; raised without antibiotics, RWA), by province (Ontario and Quebec), and by microbiota developmental path (Cluster A – Enterotype 2A or Cluster A – Enterotype 2B)

Grouping	Pre-wean ADG (g/d)^a^	Heart Girth Daily Gain (in/d)^b^	Age TP5 (d)	Age TP6 (d)	Heart girth TP5 (in)	Heart girth TP6 (in)
Overall	260.3 (82.8)	0.218 (0.092)	60 (7)	97.0 (15.0)	24.8 (3.0)	33.3 (6.8)
CONV	262.2 (89.6)	**0.238 (0.071)**	60 (7)	95.0 (20.8)	25.0 (3.4)	33.5 (8.5)
RWA	260.3 (76.9)	**0.202 (0.099)**	60 (4)	102.0 (9.0)	24.8 (2.4)	32.7 (5.1)
Ontario	**248.3 (79.2)**	**0.120 (0.121)**	59 (7)	86.0 (8.0)	24.0 (3.0)	25.0 (3.0)
Quebec	**274.1 (81.6)**	**0.228 (0.049)**	60 (3)	101 (6.25)	25.2 (2.6)	34.1 (2.9)
A – 2A	**276.0 (65.8)**	**0.220 (0.079)**	60 (4)	97 (9)	25.2 (2.1)	33.7 (3.5)
A – 2B	**233.0 (59.2)**	**0.154 (0.100)**	62 (4)	87 (11)	24.0 (1.8)	27 (4.2)

^*a*^*Pre-wean ADG* = *(BW TP3 – BW TP1)/ (Age TP3 – Age TP1)*

^*b*^*Heart girth Daily Gain* = *(HG TP6 – HG TP5)/ (Age TP6 – Age TP5)*

### Fecal sample processing

Cells in the fecal swabs were lysed using the MP FastPrep-24 5G bead-beating system. FastPrep was shaken onto the samples for 60 s at 6 m/s, then samples were spun at 8000 xg for 2 min. After this lysis step, the samples were stored at 4 °C. The DNA was extracted from the samples using MagMAX™-96 Multi-Sample Kit (Invitrogen, ThermoFisher Scientific). Diluted bead mix was washed two times and then beads were moved to an elution plate to prepare samples for 16 s rRNA PCR. Piglet fecal samples were sequenced as follows; the V3-V4 regions of 16 s rRNA genes were amplified with PCR using custom primers (forward: 5ʹ- TCGTCGGCAGCGTCAGATGTGTATAAGAGACAG-3ʹ and reverse 5ʹ- GTCTCGTGGGCTCGGAGATGTGTATAAGAGACAG-3ʹ) [21]. Clean-up of post-PCR products was carried out using Taka Bio Nucleo-Mag clean up kit (Takara Bio USA). The PCR libraries were quantified using a Qubit analysis of 8 samples per batch. A 250 base pair paired-end sequencing was performed using Illumina MiSeq platform. Mock communities (ZYMO Research International) were used to verify that DNA extraction and PCR amplification was run correctly, and environmental contamination was evaluated using negative extraction, PCR and sequencing controls.

### Sequence processing

Open-source bioinformatics software package, mothur (v1.44.3), was used to analyze DNA sequences using the mothur standard operating procedure [22]. Paired-end reads were demultiplexed and aligned to SILVA 16S rRNA reference database (version 132) to ensure that they were from the 16S rRNA V3-V4 region. Irregularities such as sequence lengths less than 442 bp or greater than 487 bp, ambiguous base calls, and long runs of homopolymers more than 8 bp were removed. Sequence data were also screened for chimeras, using VSEARCH (version 1.13.3), and non-bacterial domains (chloroplast, mitochondria, Archaea, and Eukaryotes) were removed. Remaining sequences were assigned into operational taxonomic units (OTUs) using a de novo approach based on a 97% similarity threshold (open OTU picking). Taxonomy was assigned to OTUs by aligning sequences using Ribosomal Database Project (version 9) as a reference database. OTUs that occurred in less than with less than 1% of samples were removed from the dataset.

### Bioinformatics and statistical modeling

Statistical analysis was conducted in R software (version 4.1.2). To characterize microbiota composition across early life, alpha diversity was assessed by the inverse Simpson Index with samples rarefied to 1000 sequences. Alpha diversity metrics between groups (province, rearing system, weaning status, and sampling timepoint) were compared using Whitney-Mann test or Kruskal–Wallis test. To identify patterns in fecal microbiota development, hierarchical clustering was performed using a core microbiome defined as OTUs that were present in at least 10% of samples with a minimum mean relative abundance of 0.05%. Aitchison’s distance was calculated between all samples. Beta diversity was calculated using Aitchison’s distance which was plotted on a biplot, and PERMANOVA was used to examine differences in beta diversity across timepoints. A differential abundance analysis of genera between groups was conducted using a recently developed linear regression model for compositions of microbiomes with bias correction (ANCOM-BC). The variables province, system, weaning status, and timepoint were used as covariates in the model [23]. The ward sum-of-squares algorithm was used for hierarchical clustering based on Aitchison’s distance and the optimal number of clusters was determined using gap statistics, which compared the observed change within cluster dispersion versus the expected change under an appropriate reference null distribution. After identification of appropriate number of clusters, the median age of pigs at sampling time was calculated for each cluster. Furthermore, the cluster assignment was validated by using Dirichlet multinomial mixture model (DMM) to determine if analogous clusters were created [24]. Then from the DMM, the top drivers of each cluster were compared to identify the signature genera of each cluster.

We examined differentially abundant OTUs between clusters to determine order of appearance of dominant OTUs in clusters [25]. The ANCOM-BC model also estimated the log-adjusted abundances of taxa within each sample. This adjustment includes a sample-specific sampling fraction and accounts for the compositional nature of microbiome count data, avoiding the pitfalls of false negative correlations when using relative abundance metrics in microbiome datasets [26]. Lastly, the adjusted abundances were aggregated to a phylum-level and the top 10 most abundant phyla were compared between clusters.

Finer-scale clustering was conducted within each cluster of microbiota development to identify cluster-specific enterotypes. For this step, hierarchical clustering was performed as above, but on a cluster-by-cluster basis. The distribution of pigs in each enterotype was explored based on the farm, province, and management system (whether the pigs were raised conventionally or without antibiotics). The relationship between BW and several fixed factors like age, system, province, and enterotypes present at different timepoints was examined with univariate linear mixed regression models, with sow as a random effect. These models were estimated using the R package *nlme* [27]. If factors in the univariate models had a significant effect on BW of pigs, they were combined in multivariate models, and the lowest Akaike Information Criteria (AIC) value was used to select the best-fit models. Lastly, growth performance of pigs among different microbiota succession paths were compared using PW-ADG (g/d) and G-HGDG (in/d).

## Results

### Study population

Summary data for each system, province, and cohort are displayed in Table 1. Overall, there were 9 cohorts of 40 piglets where 4 piglets were chosen from each of ten litters. Piglets were selected for this study at TP1. To the extent possible with 4 primiparous, and 6 multiparous sows, with litter sizes of 12 to 18 piglets. The mean litter size across the cohorts was 13 piglets per sow. There were 90 sows, 35 of which were primiparous, and 55 sows were multiparous. When possible, the sex ratio of the piglets was 1 male: 1 female in each cohort (total = 178 male; 182 female). Piglets from two cohorts were transported off-site for the nursery production stage.

The number of piglet sampled, their median age and standard deviation, and BW and standard deviation during each timepoint were as follow respectively: TP1 (n = 360; 4 ± 1 d; 2.1 ± 0.7 kg), TP2 (*n* = 353; 11 ± 2 d; 3.9 ± 1.2 kg), TP3 (*n *= 346; 18 ± 2 d; 6.0 ± 2.4 kg), and TP4 (*n* = 349; 27 ± 3 d; 7.2 ± 2.7 kg) (Table 1). In total, 65 pigs had at least one diarrhea occurrence from TP1 to TP4. Overall, RWA pigs had higher occurrences of diarrhea than conventional pigs. In particular, cohort 7 (RWA) had persistent diarrhea occurrences pre-weaning. Cohort 4 (RWA), cohort 6 (RWA), and cohort 9 (CONV) had 14, 7, 7 occurrences post-weaning, respectively (Table 1). There was a total of 32 samples that were not collected throughout the study. Six pigs died before TP2 and account for 18 missing samples. Four more pigs died before TP3 and account for 8 missing samples. One pig was euthanized before TP4 due to a cough. One pig was unable to be sampled at TP2 and TP3 for unknown reasons, possibly illness, and three pigs were not sampled at TP3 due to diarrhea.

### Pig performance

Median pre-weaning ADG from TP1 to TP3 was 260.3 ± 82.8 g/d (mean ± standard deviation). Notably, pre-weaning ADG was higher in Quebec pigs (274.0 ± 61.8 g/d) compared to Ontario pigs (254.0 g/d ± 64.9 g/d; Whitney-Mann Test; W = 9258; p < 0.05), but there were no differences between pigs from different rearing systems (Whitney-Mann Test; W = 12,467; p = 0.43). When comparing diarrhea occurrence in only Quebec piglets, piglets with no diarrhea had higher pre-weaning ADG (285.0 ± 62.6 g/d) than those who had pre-weaning diarrhea (285.0 ± 62.6 g/d; Kruskal–Wallis Test; chi-squared = 8.47, p < 0.05). Piglets who had post-weaning diarrhea did not have different pre-weaning ADG (268.0 ± 48.2 g/d) compared to either of the former groups. Median G-HGDG from TP5 to TP6 was 0.217 ± 0.084 in/d. The G-HGDG in conventionally reared pigs (0.24 ± 0.06 in/d) was higher than RWA pigs (0.20 ± 0.10 in/d; Whitney-Mann Test; W = 10,930, *p* < 0.001). Additionally, pigs from Quebec (0.23 ± 0.04 in/d) had higher G-HGDG than Ontario pigs (0.09 ± 0.11 in/d; Whitney-Mann Test; W = 1983, *p* < 0.001). When comparing diarrhea occurrence in only Quebec piglets, there was no difference in G-HGDG between pre-weaning diarrhea pigs, post-weaning diarrhea pigs, and no diarrhea pigs (Kruskal–Wallis, chi-squared = 0.654, *p* = 0.72).

### Sample and sequencing descriptives

The median raw read depth of 1408 sequenced samples collected from piglets at four timepoints was 27 002. Of these, 1224 samples collected from 26 to 40 pigs in each cohort were included in further analysis as 184 samples had a sequencing depth less than 1000 reads and were thus excluded (Table 1). After sequence processing, in total of 15 673 049 high quality sequences with median sequencing depth of 7650 (8942 standard deviation, SD) per sample were included in analysis. De novo clustering of sequences at 97% similarity threshold resulted in 333 406 distinct OTUs present in piglet fecal samples. The OTUs that occurred in less than 1% of sequenced samples were excluded, leading to a total of 3281 OTUs used in analysis.

### Microbiota diversity

For alpha diversity analysis, samples were rarefied to 1000 sequences per sample. Overall, the median number of OTUs observed per sample was 367.9 (182.6 SD). Overall, alpha diversity was higher in post-weaning samples (TP4) compared to pre-weaning samples (TP1, TP2, TP3) (*p* < 0.001; Table 4) and it increased from TP1 to TP4 (*p* < 0.001, Table 4). In pre-weaning pigs, there was no difference in alpha diversity (Inverse Simpson) in RWA pigs compared to CONV pigs (*p* = 0.13), nor between provinces (*p* = 0.97; Table 4). In post-weaning pigs, there was also no difference in alpha diversity (Inverse Simpson) between systems (*p* = 0.49) nor between provinces (*p* = 0.41; Table 4). At TP2, Ontario pigs had higher alpha diversity than QC pigs (*p* < 0.05), further, CONV pigs had higher alpha diversity than RWA pigs (*p* < 0.001). There were no differences in alpha diversity between province or system at any other timepoint. Based on PERMANOVA, 21.6% and 3.0% of the variation in beta diversity was explained by sampling timepoint (age of pig at sampling) and cohort, respectively. Although significant, the province and system explained less than 1% variation in beta diversity of fecal swabs.

**Table 4:** Comparison of alpha diversity (Inverse Simpson (median (SD)) of the piglet fecal swabs from different weaning statuses (pre-wean vs. post-wean), timepoints, and developmental clusters. Whitney-Mann Test and Kruskal Wallis Test were used to assess differences between groups

		Province			System		
	Overall	ON	QC	Difference	CONV	RWA	Difference
Grouping		19.1 (14.3)	19.8 (15.0)	ns	20.5 (14.1)	18.4 (15.4)	ns
Pre-wean	**15.9 (8.8)**	16.1 (9.0)	15.8 (8.6)	ns	16.1 (9.0)	15.5 (8.5)	ns
Post-wean	**34.4 (16.7)**	35.3 (16.3)	33.7 (17.0)	ns	33.7 (16.1)	36.2 (17.4)	ns
	***p* < *0.05***						
Visit 1	**12.6 (5.4)**	12.7 (4.5)	12.6 (6.1)	ns	12.5 (4.9)	13.4 (6.1)	ns
Visit 2	**16.7 (8.8)**	**18.1 (9.6)**	**15.0 (7.9)**	***p* < *0.05***	**18.9 (9.3)**	**14.8 (7.6)**	***p* < *0.05***
Visit 3	**21.1 (9.6)**	21.5 (9.8)	20.9 (9.5)	ns	22.0 (9.3)	19.7 (10.0)	ns
Visit 4	**34.4 (16.7)**	35.3 (16.3)	33.7 (17.0)	ns	33.7 (16.1)	36.2 (17.4)	ns
	***p* < *0.05***						
Cluster A	**12.5 (5.2)**	12.6 (4.7)	12.3 (5.7)	ns	12.1 (4.6)	13.3 (5.9)	ns
Cluster B	**18.5 (9.3)**	19.7 (9.5)	17.6 (9.1)	ns	20.0 (9.3)	16.7 (9.1)	***p* < *0.05***
Cluster C	**34.9 (16.7)**	38.3 (16.3)	33.7 (17.0)	ns	33.8 (16.2)	36.6 (17.3)	ns
	***p* < *0.05***						

### Microbiota composition

**Fig. 1:**
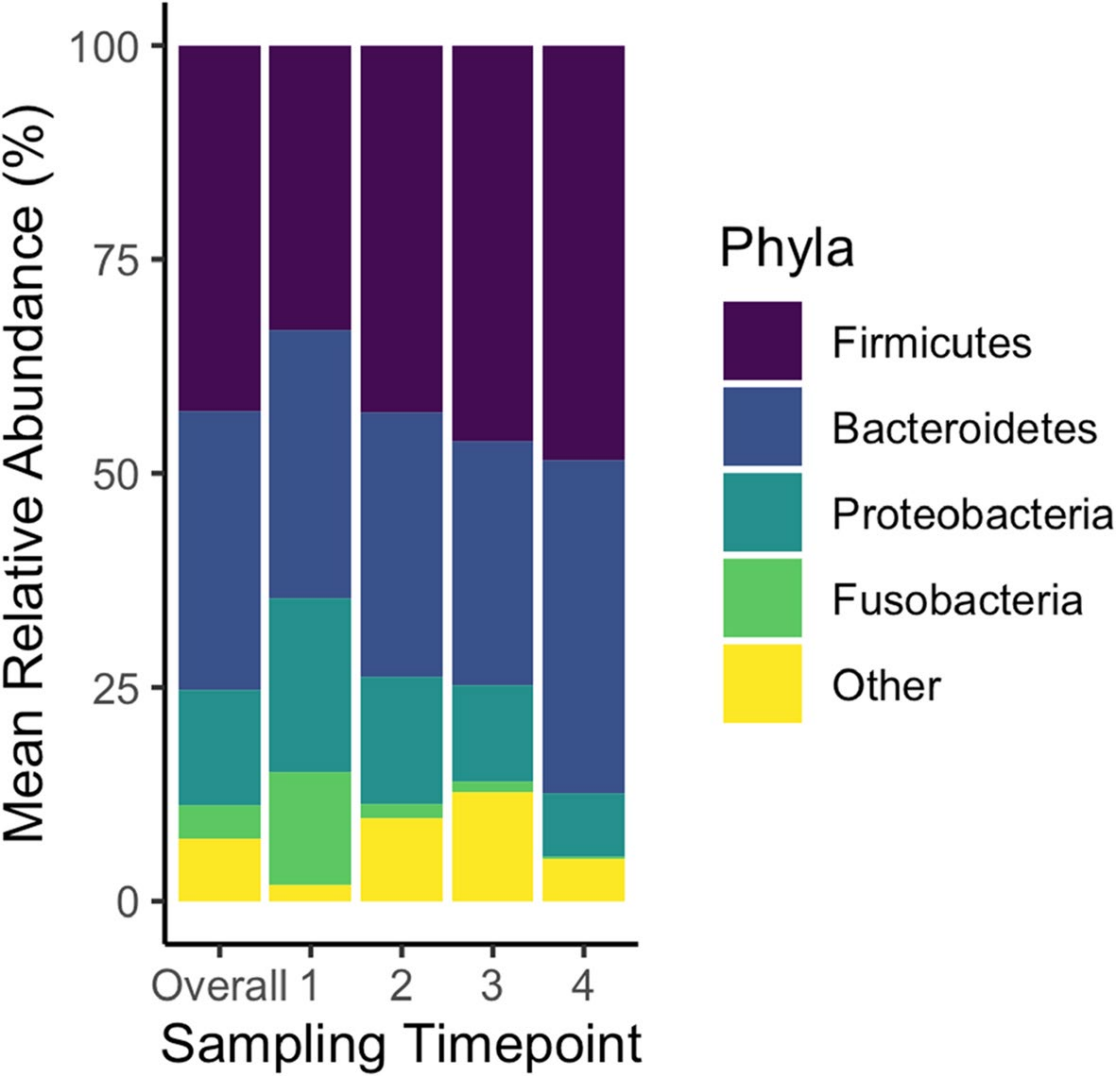
Mean relative abundance of phyla overall across all timepoints, and by timepoints 1, 2, 3, and 4

Across all 1224 samples from four timepoints, seven phyla with a mean relative abundance over 1% (accounting for 97% of the sequences) were identified including Firmicutes (mean relative abundance ± standard deviation = 42.8% ± 12.5%), Bacteroidetes (32.6% ± 11.3%), Proteobacteria (13.4% ± 13.0%), Fusobacteria (4.0% ± 7.2%), Synergistetes (1.7% ± 4.0%), Spirochaetes (1.3% ± 3.2%), and Verrucomicrobia (1.3 ± 4.2%) (Fig. 1**)***.* The top 10 families (accounting for 78% of sequences) were *Bacteroidaceae*, *Prevotellaceae*, *Lachnospiraceae*, *Ruminococcaceae*, *Enterobacteriaceae*, *Acidaminococcaceae*, *Porphyromonadaceae*, *Fusobacteriaceae*, *Lactobacillaceae*, and *Veillonellaceae* (Table S2, Additional File 2). Of 186 genera identified, four genera occurred with mean relative abundance over 5% including *Bacteroides*, *Prevotella*, *Escherichia/Shigella*, and *Phascolarctobacterium*, and accounted for 35% of the total sequences. Further, 10 genera and one group had a mean relative abundance above 1% across all timepoints including the group *Clostridium* cluster *XIVa*, and genera *Lactobacillus*, *Fusobacterium*, *Veillonella*, *Barnesiella*, *Cloacibacillus*, *Treponema*, *Akkermansia*, *Butyricimonas*, *Megasphaera*, and *Streptococcus* (Table S2, Additional File 2).

**Fig. 2:**
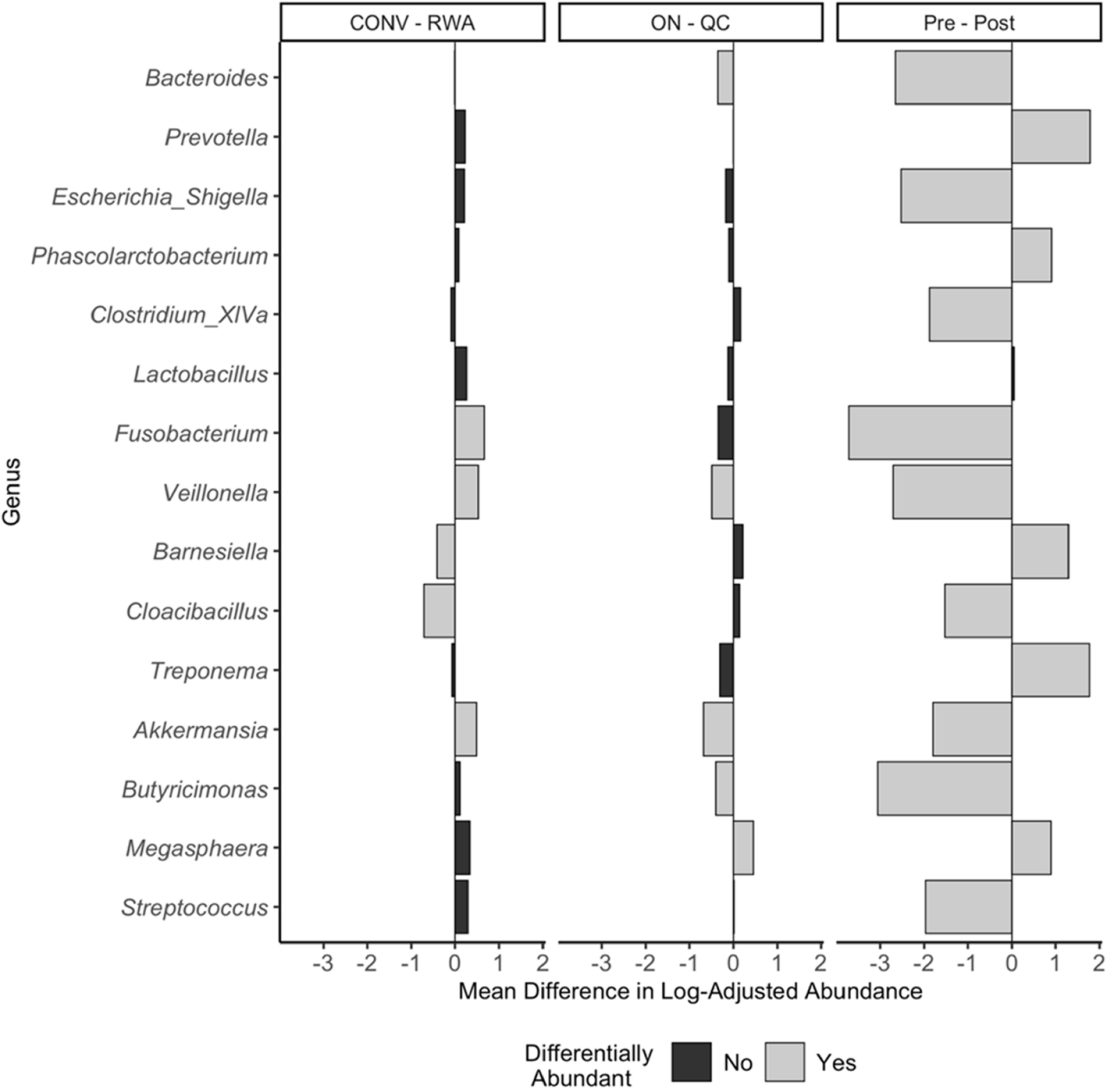
Differentially abundant genera between rearing system (RWA vs. CONV), province (ON vs QC), and weaning status (pre-weaning vs post-weaning). The top 15 most abundant (descending) genera across all timepoints are displayed and significant differences in abundance indicated by bar colour. Bar height represents magnitude of difference

**Fig. 3:**
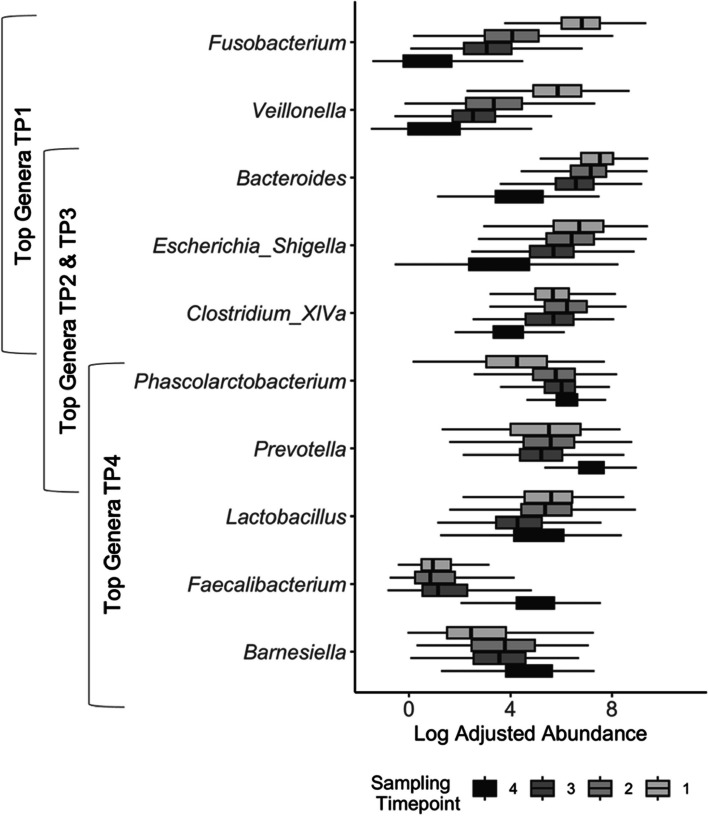
Log-adjusted abundances of the top 5 genera at sampling timepoint (TP) 1, 2, 3, and 4. There is no overlap between the top 5 genera at TP1 and TP4. All genera presented are differentially abundant between at least two visits

The ANCOM-BC was implemented at the genus level to identify differentially abundant genera between groups. Results of ANCOM-BC for the top 15 genera are shown in Fig. 2. After accounting for sampling timepoint and weaning status, when comparing pigs raised conventionally to RWA pigs, 28 genera were differentially abundant. Among the top 15 genera, *Fusobacterium*, *Veillonella*, and *Akkermansia* were more abundant in RWA pigs, while *Barnesiella*, and *Cloacobacillus* more abundant in conventionally raised pigs (Fig. 2). Comparing pigs from different provinces, 64 total genera were identified as differentially abundant with *Bacteroides*, *Veillonella*, *Akkermansia*, and *Butyricimonas* being more abundant in ON pigs and *Megasphaera* more abundant in QC pigs. When comparing the microbiota of pre-weaned pigs with post-weaned pigs, there were 117 genera that were differentially abundant, indicating a large shift in composition of post-weaning samples. All top 15 genera showed significant differences in abundance with the exception of *Lactobacillus* (Fig. 3). When comparing genera across sampling timepoints, 125 genera were identified as differentially abundant between at least two timepoints. Further, *Bacteroides*, *Escherichia/Shigella*, and *Clostridium* cluster *XIVa* were the top five genera for TP 1, 2, and 3; whereas the genera *Phascolarctobacterium* and *Prevotella* were within the top five for TP 2, 3, and 4 indicating that those genera might dominate the microbiota later during nursery stage (Fig. 3).

### Hierarchical clustering

**Fig. 4:**
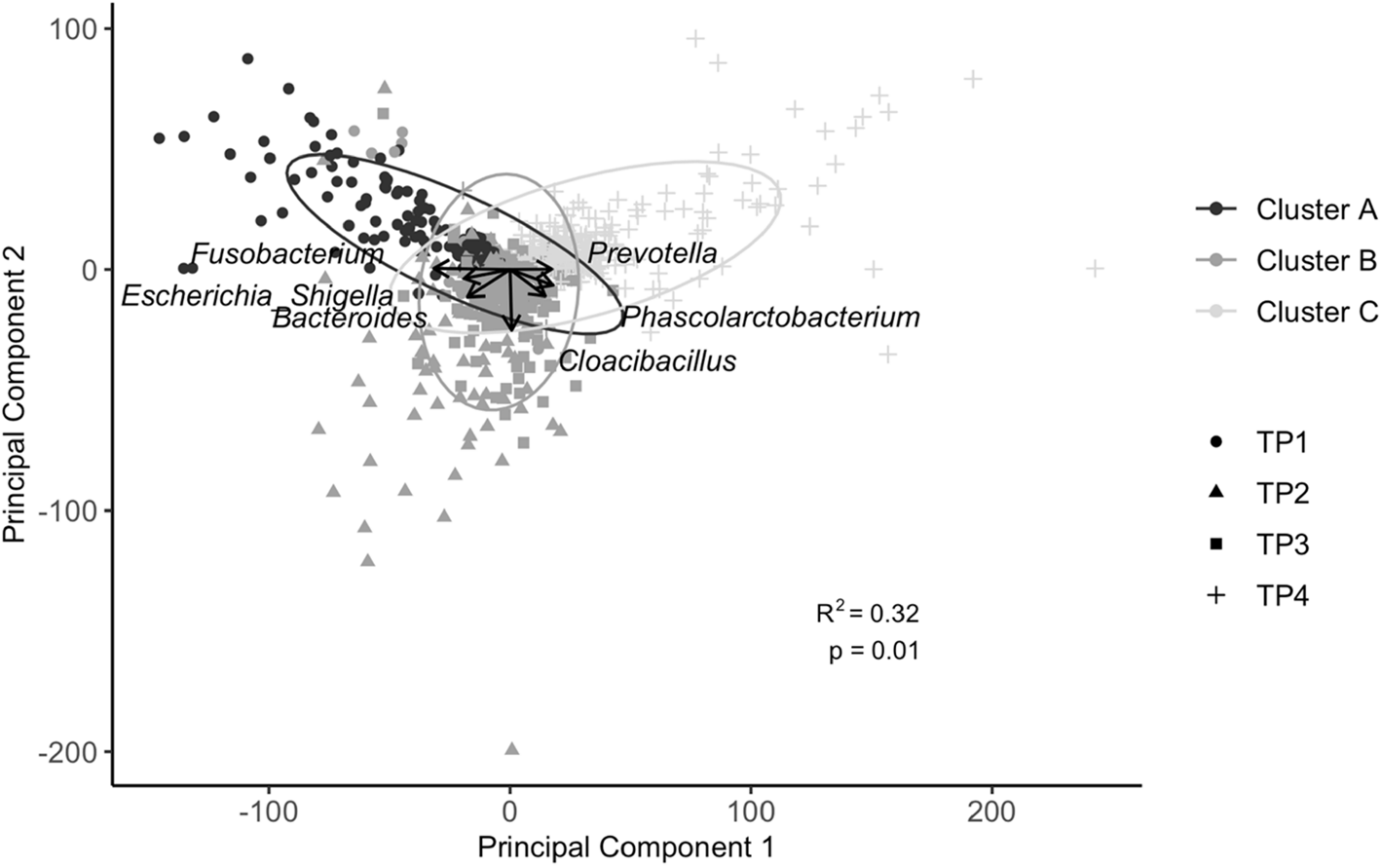
Biplot displaying beta diversity (Aitchison’s Distance) between samples. Samples are shaded by their cluster assignment, and the shape of the points reflects the sampling timepoint. Almost all samples from timepoint 1 are classified in Cluster A, samples from timepoint 2 and 3 are in Cluster B, and samples from timepoint 4 are in Cluster C. Principal component 1 explains 26.4% of variation in beta diversity, and principal component 2 explains 10.6% of variation in beta diversity. Loadings of the top 7 genera are displayed with arrows on the PCA

Using Aitchison’s distances and gap statistics three distinct clusters (A, B, and C) were identified based on beta diversity of core OTUs (Fig. 4). Cluster A and Cluster B were mostly identified in pre-weaned pigs, while Cluster C contained almost all post-weaning pigs. Furthermore, Cluster A contained most samples collected at TP1, while Cluster B contained samples collected at both TP2 and TP3 (Fig. 4). Pigs from all 9 cohorts, regardless of rearing system, province, or sex followed the same successive stages across early life (Fig. 4**)**. Alpha diversity (Inverse Simpson) increased from cluster A to B to C (Table 4**,** KW; *n* = 1224, *p* < 0.001). At the genus level, beta diversity (Aitchison’s Distance) was significantly different between clusters (Fig. 4).

There were 2762 OTUs identified as differentially abundant between clusters. Of the top twenty most abundant OTUs, 17 were identified as differentially abundant (Figure S2**, **Additional File 3). OTUs that were not differentially abundant between the three clusters were Otu000017 and Otu000035 which were both Unclassified *Lachnospiraceae*, and Otu000055 from the genus *Megasphaera* (Figure S2**, **Additional File 3)*.* Examination of the PCA plot (Fig. 4) showed samples in cluster A had higher levels of the genera *Fusobacterium*, *Escherichia/Shigella*, and *Bacteroides* which was reflective of the genus-level composition in samples collected at TP1. Further, *Cloacibacillus* was elevated in Cluster B while *Prevotella*, *Phascolarctobacterium*, and *Barnesiella* are more abundant in Cluster C.

To validate the successive stages identified with a different method, the DMM model was used for determining community types within the samples, and analogous results were found. Three clusters were identified using DMM model with 93% (1149/1224) samples following the same pattern as seen with hierarchical clustering. The top community drivers of each cluster, as identified by DMM modelling, are shown in Figure S1 (Additional File 3). Cluster A was driven by the abundance of *Escherichia/Shigella*, *Bacteroides*, *Fusobacterium*, *Phocaeicola*, *Veillonella*, and *Clostridium*; Cluster B was driven by Unclassified *Ruminococcaceae*, *Bacteroides*, Unclassified *Lachnospiraceae*, *Phascolarctobacterium*, *Escherichia/Shigella*, and *Prevotella*; and Cluster C was driven by *Prevotella*, Unclassified *Ruminococcaceae*, *Phascolarctobacterium*, Unclassified *Lachnospiraceae*, Unclassified *Bacteroides*, and Unclassified *Clostridiales* (Figure S1, Additional File 3).

### Microbiota enterotypes

**Fig. 5:**
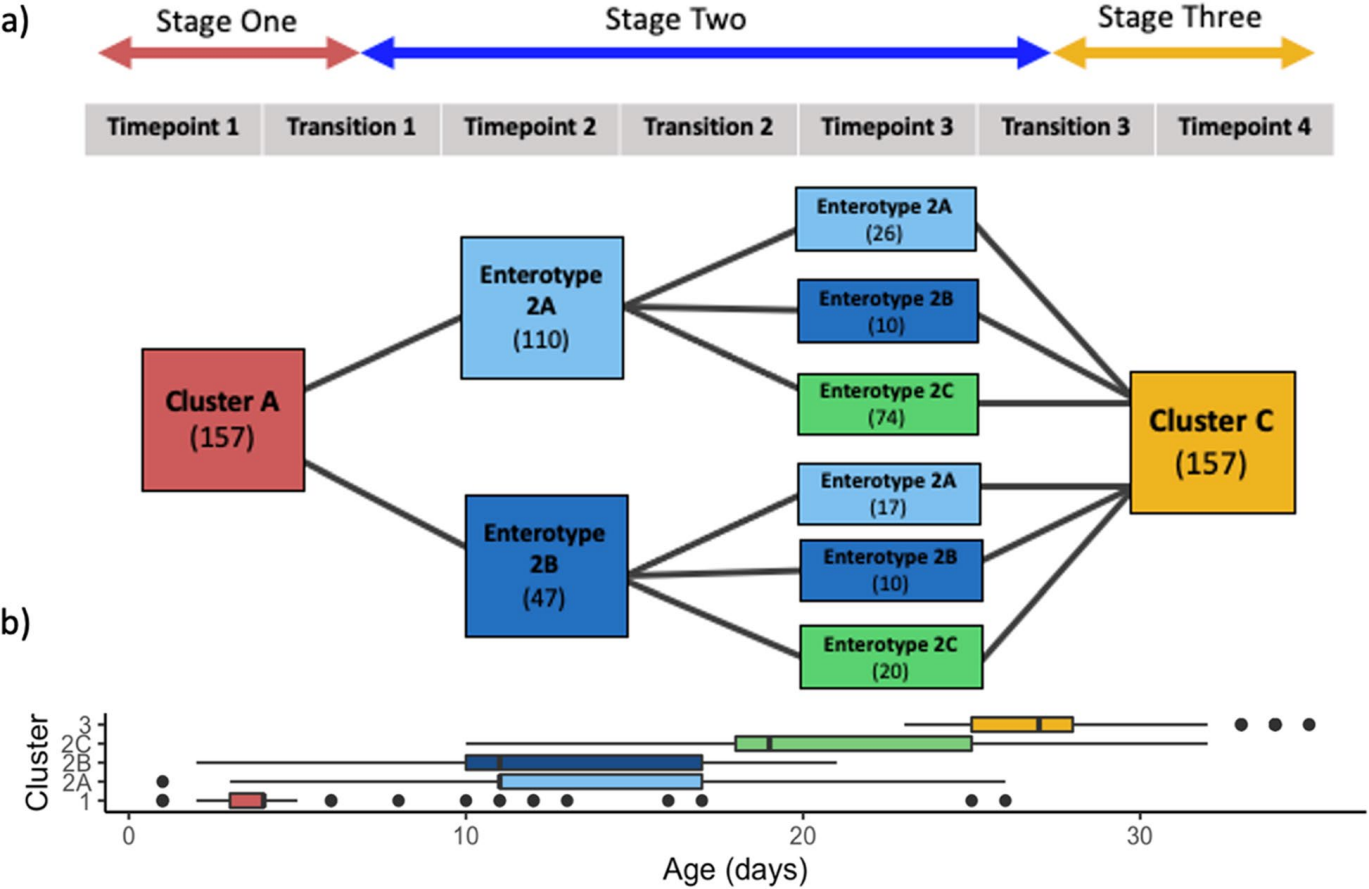
**a** Succession paths observed in 157 piglets across timepoint 1, 2, 3, and 4. In each box, the cluster (A, B, C) or enterotype (2A, 2B, 2C) is shown with number of pigs who follow that respective path, **b** distribution of the piglets’ age within clusters and enterotypes

**Fig. 6:**
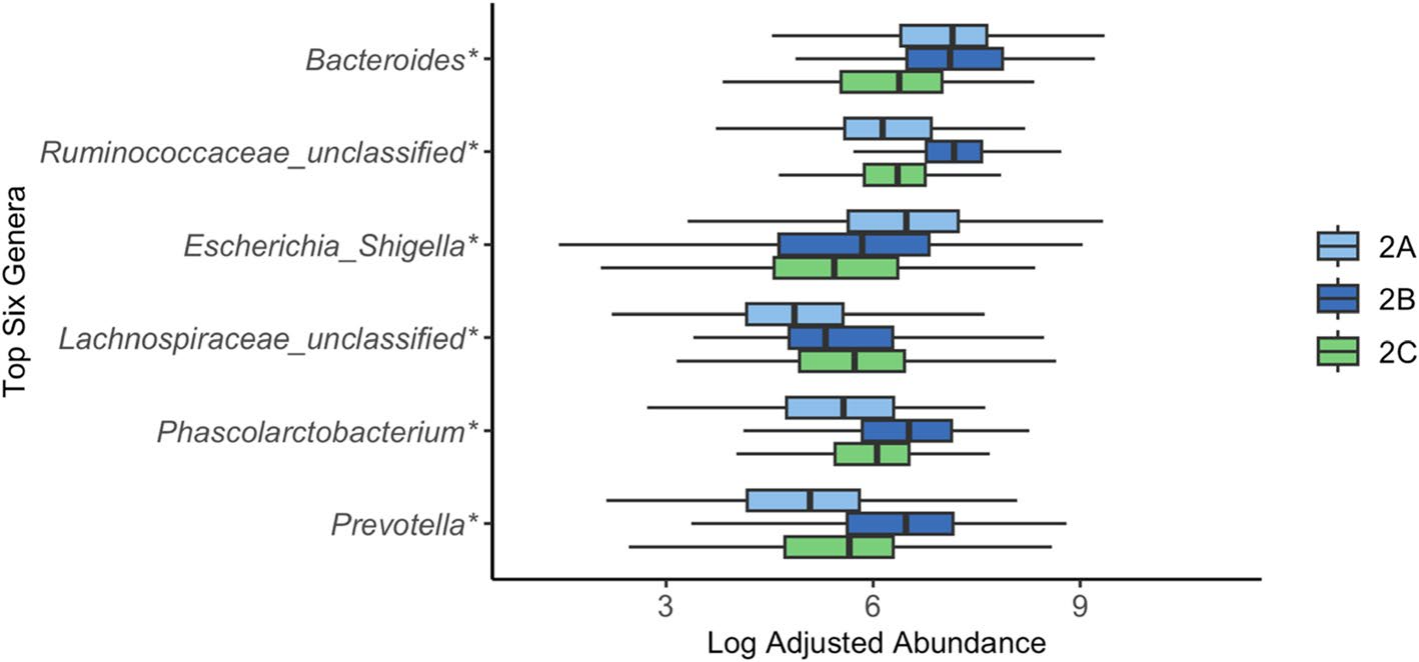
Log-adjusted genus abundance estimates by ANCOM-BC from Cluster B samples (*n* = 622) in each of the enterotypes (2A, 2B, 2C). Top six most abundant genera in Cluster B are displayed with asterisk (*) indicating genus differential abundance between enterotypes

To identify microbiota enterotypes, the samples within each of the three clusters were split into hierarchical enterotypes using the core 269 OTUs. Gap statistics indicated that there were no enterotypes identified in Cluster A and Cluster C. However, in Cluster B (*n* = 622) three distinct enterotypes were identified including Enterotype 2A (*n* = 296), 2B (*n* = 139), and 2C (*n* = 187). To trace the succession patterns in pigs, a total of 157 pigs were used that had samples collected from TP1, TP2, and TP3 (Fig. 5). There are two common succession pathways from TP1 to TP2; 110 pigs started in Cluster A at TP1 and then moved to Enterotype 2A, and 47 pigs started in Cluster A and moved to Enterotype 2B during TP2 (Fig. 5). There six major pathways from TP1 to TP3, the most common path was Cluster A to Enterotype 2A to Enterotype 2C (*n* = 74). Furthermore, Enterotypes 2A and 2B appeared in samples from TP 2 (median sampling age = 11 days) and TP 3 (median sampling age = 18 days). Enterotype 2C occurred only at sampling TP 3 (Fig. 5). In the Quebec pigs, all RWA, and 75% of CONV pigs were classified into Enterotype 2A at TP2. In the Ontario pigs 50% of RWA pigs, and 50% of CONV pigs were classified into Enterotype 2A at TP2. At TP 3, regardless of province and rearing system, the dominant enterotype was Enterotype 2C. Interestingly, Enterotype 2B was not present in conventionally raised Quebec pigs. ANCOM-BC identified a higher abundance of unclassified *Ruminococcaceae*, *Prevotella*, and *Phascolarctobacterium* in Enterotype 2B compared to the other enterotypes, while Enterotype 2C had a lower abundance of *Escherichia/Shigella* and *Bacteroides* compared to the other enterotypes (Fig. 6). Unclassified *Lachnospiraceae* was not differentially abundant between enterotypes (Fig. 6).

### Factors associated with performance

**Fig. 7:**
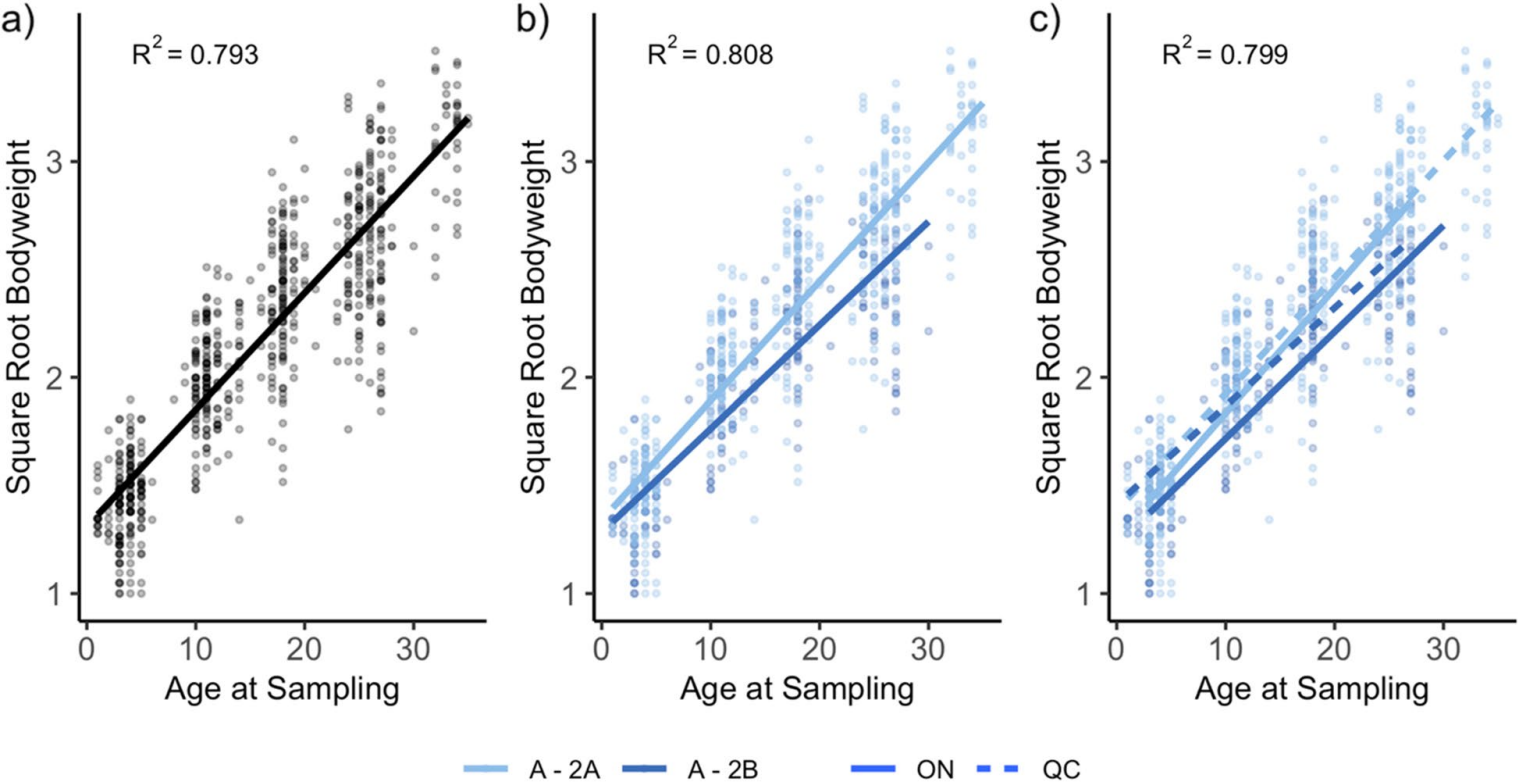
Linear regression models for the body weight of the pig at time of sampling from the top three models identified (n = 628 samples; n = 157 pigs). Variables in models are a) age of pig, b) age of pig and timepoint (TP)1 – TP2 progression pathway, and c) age of pig at sampling, TP1 – TP2 progression pathway, and province

Univariate linear regression models tested the fixed variables age, TP1—TP2 succession path, TP1—TP3 succession path, production system, pre-weaning diarrhea occurrence, and province to determine the association with bodyweight. All fixed effects were significant in univariate models except production system and diarrhea occurrence (Table S3, Additional File 3). Multivariate models were constructed using significant factors (Table S3, Additional File 3). The top three models identified by AIC values were graphed to show the effect of age alone (Fig. 7a), age and TP1-TP2 succession path (Fig. 7b), and all of age, TP1-TP2 succession path, and province (Fig. 7c) on BW. Interestingly, when considering TP1 – TP2 progression, pigs who follow succession pattern Cluster A – Enterotype 2A had BW increase at a higher rate, shown by the slope of the fitted line. This was further explored by comparing pre-weaning ADG between the top two TP1 – TP2 progression patterns. Pigs with Cluster A – Enterotype 2A pattern had higher pre-weaning ADG (Whitney-Mann; n = 157, *p* = 0.00029; Table 3), and G-HGDG (Whitney-Mann; *n* = 157, *p* = 1.9 × 10^–5^; Table 3) than pigs who progressed Cluster A – Enterotype 2B.

## Discussion

The purpose of this study was to characterize the changes in pig fecal microbiota from birth until one-week post-weaning, analyze its diversity and composition, and its relationship with growth performance. The study cohorts were in Ontario or Quebec and raised either without antibiotics or under a conventional rearing system which used antibiotics such at chlortetracycline, lincomycin, aureomycin, and aivlosin (Table S1, Additional File 1). At TP2, CONV pigs had higher alpha diversity than RWA pigs and Ontario pigs had higher alpha diversity than Quebec pigs. However, at all other sampling time points, alpha diversity was not different between provinces, or rearing systems. This may be explained by first dose of antibiotics in CONV piglets being administered during lactation, coinciding with TP2. In response to antibiotics, the abundance of targeted bacteria decreases, typically lowering alpha diversity [18]. This response is not long lasting, as there was no difference in alpha diversity at TP3, or TP4. It is possible that antibiotics decrease target bacteria which then opens up new niches for common commensal gut bacteria like *Escherichia*, *Lachnobacterium*, *Clostridium*, and *Aerococcus* which have increased in abundance after antibiotic treatments in previous studies [28–30] In CONV cohorts, the second dose of antibiotics (if administered) occurred post-weaning, in the nursery stage, after sampling of the piglets was complete. *Akkermansia* was among the few genera that had a higher abundance in RWA pigs. This is a beneficial bacterium that degrades mucus and it plays a role in colonization resistance to pathogens that must cross the mucus layer to invade epithelium cells [31]. Previous studies have shown antimicrobial use having significant impacts on gut microbiota diversity [21, 22] and the incidence of antibiotic resistant genes within the microbiota are commonly elevated when antibiotics are used [24]. It is possible that different types of antibiotics were used on conventional farms and impacts to microbiota may depend on the antibiotic administered, or administration method [21, 25]. Only one farm reported administering antibiotics in water as opposed to in-feed antibiotics. Furthermore, differences between rearing conventions may be less prominent in young piglets as the sample were taken in early life, prior to antimicrobial exposure. Based on beta diversity, the farm explained 3% of overall diversity, indicating that different rearing practices only minimally impacted overall microbiota composition. This may be due to the large number of differing variables between farms, as shown by the farm surveys (Table S1**, **Additional File 1). A previous study of microbiota in one to two week old piglets found similar results, suggesting that housing had a small effect size although was significantly associated with microbiota composition [11]. Kubasova and colleagues however did not find any association between housing factors and microbiota of piglets, but did find such associations with sow microbiota [26]. It is possible that environmental differences manifest in more mature microbiota in adults as opposed to microbiota in young piglets.

In this study fecal microbiota was analyzed at four timepoints from a few days after birth until one-week post-weaning during which the GI tract might have undergone several structural and physiological changes. This may explain the changes in alpha diversity and beta diversity observed across sampling timepoints in the current study. Some studies have shown differing results relating to alpha diversity and age of pig, however, most, like the present study, reported increasing alpha diversity with age [32, 33]. Based on the sequencing approach used in this study, it is not possible to deduce the absolute abundance of microbes within the gastrointestinal tract, however, the gut microbiota in early life may increase in diversity as the pigs become exposed to more microbes [34]. As pigs age, they begin receiving less maternal antibodies that prevent microbial colonization, and as this occurs, a higher diversity of microbes may colonize the GI tract explaining the increased alpha diversity observed here [35]. Further, sampling timepoint was shown to explain 21.6% of the variation in beta-diversity suggesting that the age of the pig at sampling is key in understanding the dynamics of the microbiota. Age may play an important factor because there are physiological changes in gut structure as a pig matures, so pigs of similar ages would have similar gut environments and thus support similar microbiota communities [9].

Similar to other studies, *Firmicutes*, *Bacteroidetes,* and *Proteobacteria* were the dominant phyla in piglet microbiota [36]. However, the phylum *Fusobacteria* dominated microbiota at TP1, but was almost absent at all other timepoints. In fact, the genera *Fusobacterium* and *Veillonella* (both the phylum Fusobacteria) were unique to the profile of TP1. *Fusobacterium* is usually high in the suckling gut microbiota [32, 37]. Many bacteria in the phylum *Fusobacteria* are pathobionts, meaning they await opportunities to become pathogenic. Pathobionts are commonly found in early piglet gut microbiota as the community is not diverse and beneficial bacteria that outcompetes pathobionts has not colonized [4, 32]. *Veillonella* is commonly found in suckling piglets as it is known for lactate fermentation [38]. Timepoints 1, 2, and 3 all contained *Bacteroides, Escherichia/Shigella,* and *Clostridium* cluster XIVa in their top five genera which are all commonly commensal bacteria in fecal microbiota. *Prevotella* was present at TP2 and 3, but it dominated the microbiota at TP4 when all samples are taken from weaned piglets whose diet consists of solid feed. At weaning, the functional capacity of the microbiota shifts from milk glycan metabolism to plant glycan metabolism [17]. *Prevotella* is a common, and beneficial microbe that metabolizes complex starch molecules [39], and it’s influx reflects the shift that occurs in the type of substrate available in the gut. [4, 17]. In the current study, two cohorts received creep feed starting at 5 days, two at 10 days, and three at 18–20 days of age. This may explain the slight increase in *Prevotella* in pre-weaned pigs at TP2 and 3 as starch substrate was available in the gut from the creep-feed ingredients. The genus *Faecalibacterium* was also elevated in weaned piglets (TP4). *Faecalibacterium* has been found to be a beneficial bacteria that is associated with later-weaned piglets [40]. Further, although *Lactobacillus* is generally characteristic of a milk-oriented microbiome [41] and is expected to decrease in the weaned pigs [32, 34], it was one of the few genera that was not differentially abundant between sampling timepoints. This may indicate that there is a gradual adjustment in the abundance of *Lactobacillus* after weaning, as levels of *Lactobacillus* one-week post-weaning were not different from the abundance in pre-weaned pigs. Post-weaning diarrhea is a major concern at this transition and into the nursery stage. Wang and colleagues found it takes between seven to nine days for the microbiota to stabilize after weaning leaving piglets vulnerable to pathogen invasion [17]. Weaning is one of the most important transitions throughout production stages and further research on how microbiota responds to this stress is needed before interventions that focus on microbiota as a therapeutic target to prevent post-weaning diarrhea can be implemented.

There were three major clusters of microbiotas identified in fecal samples collected from pigs at different timepoints. Cluster A occurred from birth until one week of age, followed by Cluster B from one week of age until weaning, and Cluster C was comprised of the post-weaning samples from TP4. This distribution of samples in clusters allows for the identification of three major stages of microbiota succession seen across the early life of piglets. Cluster B is comprised of samples from both TP2 and TP3, suggesting that piglet microbiota from 11 days of age until one week post weaning is relatively homogenous and compositional differences across these timepoints are less pronounced. As expected, there was a distinct shift in microbiota composition between the suckling and nursery stage largely driven by weaning which was also found in a recent meta-analysis conducted by Luo et al. [16]. The differences seen between clusters are reflective of the progression of the microbiota as piglets get older with *Fusobacterium* being a driver of Cluster A, and *Prevotella* driving Cluster C [16]. The three microbiota clusters were identified in all fecal samples collected from pigs in both provinces and production rearing system, suggesting that this pattern might be widespread amongst pigs. Explicit clusters and corresponding composition illustrate widespread microbial succession patterns in the fecal microbiota of young piglets.

Three enterotypes 2A, 2B, and 2C were identified in the fecal microbiota of pre-weaned pigs older than 7 days. These enterotypes are practical clusters of community-types that may cause similar outcomes across pigs with the same enterotype [42]. Several grow-finish enterotypes in swine have been identified in previous studies [18, 20, 43]. Enterotypes are usually age-dependent and piglet neonatal enterotypes have not been reported [44]. In this study no enterotypes were identified in the microbiota of fecal samples collected from pigs younger than one week old, or from post-weaning pigs. However, within Cluster B (pre-weaned pigs older than 7 days), three enterotypes (2A, 2B, and 2C) were identified. Enterotype 2C occurred only at TP3, indicating that it may be associated with later weaning. Piglets from both provinces and two different rearing system had high occurrences of Enterotype 2C at TP3 indicating that it is a dominant community type at this stage. No pigs from conventional farms in Quebec exhibited Enterotype 2B at TP3. When examining the top 6 genera, Enterotype 2C had lower levels of *Bacteroides* and *Escherichia/Shigella*. Several bacterial serotypes of *Escherichia/Shigella* are opportunistic pathogens [45]. During early days of post-weaning, the gut is in dysbiosis and may be vulnerable to the pathogenicity of these bacteria [46]. Piglets in Enterotype 2C had a lower abundance of this genus and may have been less vulnerable to their pathogenic effects. It is not possible to discern the serotype of a bacteria when using 16S rRNA gene sequencing, so further exploration of the associations between early microbiota and productivity are needed to explain these differences in performance. It has been previously demonstrated that enterotypes are not absolute during the nursery stage [16]. Identification of enterotypes suggests microbiota composition during the two-week pre-weaning period might not be stable, and in contrast to a stable adult microbiota, it may be more easily influenced by preventative treatments and therapeutics. This is also seen in humans where microbiota in infancy has a high plasticity and is reactive to changes in external environments, so this stage is being used as an opportunity for external intervention to promote health [44]. Studies have been conducted on identifying enterotypes in growing and finishing pigs, and two enterotypes have been identified in multiple studies [4, 28, 33, 47]. In all studies, one cluster is associated with *Prevotella*, while the second cluster is associated with different genera such as *Clostridium* [4], *Ruminococcus* [47], or *Treponema* [28, 33]. This suggests that antimicrobial alternatives may be used in piglets within the two-week pre-weaning period to promote beneficial microbial succession.

There were associations between pig productivity and succession pathways in pigs. When comparing succession paths from TP1 – TP2, pigs who exhibited Enterotype 2A at TP2 had higher performance than those who exhibited Enterotype 2B at TP2. Enterotype 2A had lower levels of Unclassified *Ruminococcaceae*, *Prevotella*, and *Phascolarctobacterium* genera. Previous studies on grow-finish pigs enterotypes, have shown the enterotype dominated by *Prevotella* has a higher productivity metrics compared to other enterotypes [47, 48]. *Phascolarctobacterium* and *Prevotella* are often characterized as commensal in the gut, however in this study, the enterotype with a lower abundance of these genera was positively associated with growth performance. As gut microbiota is a complex ecosystem, the community network may determine functional differences between enterotypes that lead to differences in performance, rather than performance being associated with one individual genera [47]. The effect of gut microbiota on growth observed in this study is consistent with previous findings that gut microbiota in early life may have long-term impacts on gut community structure, which may in turn be causing differences in performance [11]. Further research is needed in this area to delineate bacteria or community networks which increase productivity in early life, based on these results, optimal microbiota development may follow a succession where Enterotype B is not encountered.

## Conclusions

In this study, the development of pig microbiota was explored from birth to one-week post-weaning. Pig microbiota underwent three successive stages across this time. Each stage is associated with differences in community structure on a phylum-, genus-, and OTU-level. Large scale microbial variation at stage two can be accounted for using three enterotypes that are also associated with productivity of the pig at three different times: pre-weaning, post-weaning, and in the suckling phase. Microbiota of pigs in stage two was shown to shift from one enterotype to another, meaning it may be responsive to alternative therapeutics. This study highlights major developmental changes in swine microbiota, identifies periods of microbiome shifts, and relates early microbiota to pig growth. Understanding the dynamics of microbial succession in early life, and its impact on health and growth, are preliminary steps in introducing prebiotics, probiotics, postbiotics, and phytogenics to optimize swine health.

### Supplementary Information

Supplementary Material 1.

Supplementary Material 2.

Supplementary Material 3.

## Data Availability

The datasets presented in this study can be found in online repositories. The names of the repository/repositories and accession number(s) can be found at: https://www.ncbi.nlm.nih.gov/bioproject/PRJNA1001900

## Abbreviations

AIC: Akaike Information Criterion
ANCOM-BC: Analysis of compositions of microbiomes with bias correction
bp: Base pair
clr: Center-log ratio
CONV: Conventional rearing system
DMM: Dirichlet multinomial mixture
DNA: Deoxyribonucleic acid
g: Grams
GI: Gastrointestinal
G-HGDG: Growing stage heart girth daily gain
in: Inches
kg: Kilograms
KW: Kruskal-Wallis
ON: Ontario
OTU: Operational taxonomic unit
PCR: Polymerase chain reaction
PERMANOVA: Permutated analysis of variance
PW-ADG: Pre-weaning average daily gain
QC: Quebec
RDP: Ribosomal database project
rRNA: Ribosomal ribonucleic acid
RWA: Raised without antibiotics
TP: Timepoint
