# Supplementary material for: Progression of swine fecal microbiota during early stages of life and its association with performance: a longitudinal study
Source: BMC Microbiol. 2024 May 25;24:182. doi: 10.1186/s12866-024-03336-y (PMC11127378; doi:10.1186/s12866-024-03336-y)
Supplement: Supplementary file 1 — Supplementary Material 1. [file 12866_2024_3336_MOESM1_ESM.pdf]

Additional file 1. Farm survey data from nine cohorts in this study.

**Cohort**	**1**	**2**	**3**	**4**	**5**	**6**	**7**	**8**	**9**
**Province**	ON	ON	ON	ON	QC	QC	QC	QC	QC
**System**	conventional	rwa	conventional	conventional	rwa	rwa	rwa	conventional	conventional
Farm Size									
**Sow**	1040	600	-	350	400	240	155	540	500
**Nursery**	2953	2000	-	650	2000	900	475	1100	1760
**Finisher**	8526	5000	-	150	2000	1235	2000		6000
**Replacement Gilts**	126	50	-	30	120	10	10	40	40
**Suckling**	1948	1000	-	150	1000	480	475	1050	1200
**Grower**		-	-	450	3000	865		-	-
**Boars**	15	8	-	4	3	2	2	1	4
**Farrow Spaces**	200	108	-	-	94	42	48	105	195
**Farrow Rooms**	3	5	-	6	9	6	3	-	-
**Dry Sow Spaces**	800	528	-	6	-	198	140	364	350
**Boar Spaces**	15	8	-	1	-	2	2	1	4
**Nursery Spaces**		-	-	-	-	560	456	1100	1760
**Nursery Rooms**	14	8	-	7	6	7	-	-	-
**Finisher Spaces**	-	2 barns x 12 pens x 6 sorters	-	-	-	-	2004	1100	6000
**Finisher Rooms**	36		-	4	2-3 per barn	7			
**Gilt Spaces**	180	-	-	-	4	-	10	90	35
**Gilt Pens**	-	3	-	-	-	-	-	-	-
**Gilt Rooms**	-	-	-	-	-	1	-	-	-
**Isolation Spaces**	-	-	-	-	-	-	-	-	70

Farm Metrics									
**Live Animal Entry**	no	yes		no	yes	no	yes	yes	yes
**Live Animal Type**	-	gilts	-	-	gilts, boars	-	gilts, boars	gilts, boars, nursery, grow-finish	gilts
**Quarantine Barn**	no	no	-	-	no	-	yes	yes	yes
**Multi-site Operation**	yes	yes	-	no	yes	no	yes	yes	yes
**Number of Sites**	4	4	-	-	8	-	4	3	4
**Number of breeding sites**	-	1	-	-	1	-	1	1	1
**Number Nursery Sites**	-	1	-	-	2	-	1	1	1
**Number grow-finish sites**	-	2	-	-	5	-	2	1	2
**Number of nursery-grow-finish sites**	-	-	-	-	-	-	0	0	-
**Animal Sourcing**	-	single	-	-	single	single	single	single (farrowing), multi(nursery)	single
**Continuous Flow**	yes	yes	-	yes	yes	yes	yes	yes (farrowing), no (nursery and finishing)	yes
**Barn - all in all out**	no	-	-	no	-	no	no	no	no
**Breeding - all in all out**	no	-	-	no	no	no	no	no	no
**Farrowing - all in all out**	yes	yes	-	yes	yes	yes	yes	yes	yes
**Nursery - all in all out**	yes	yes	-	yes	no	yes	yes	yes	yes
**Grower - all in all out**	yes	-	-	no	yes	no	yes	yes	-
**Finisher - all in all out**	yes	no	-	no	yes	no	yes	yes	yes
**Target market weight**	123.377	109	-	-	130	125-135	135		130
**Target market age**	16		-	-	26	26	24	26	25
**Source of replacement gilts**	single	multi	-	single	multi	single	single	multi	multi

**Source of boar**	0	multi	-	single	multi	single	single	multi	single
**source_semen**	0		-	multi	multi	multi	single	multi	multi
**#_pigs_sow_year**	-	29.3	-	17.8	26.8	27.94	27.8	26.62	26.3
**litter_size**	-	13.4	-	12.7	15	13.7	14.08	12.68	13.12
**preweaning_mortality**	-	-	-	6.80%	18.7	15.9	14.5	12.34	15.52
**postweaning_mortality**	-	-	-	-	3	-	-	-	-
**nursery_growfinish_mortality**	-	-	-	-	4%	<5%	4%	4-5%	6%
**days_to_market**	-	-	-	-	190	192	163	182	175
**Average market weight (kg)**	-	109	-	-	135	125	135	125	130
**Shower Out**	yes	no	-	yes	no	no	no	no	no
**Shower In**	yes	no	-	yes	no	no	no	yes	yes
**All In**	no	yes	-	no	yes	-	yes	yes	yes
**All Out**	-	yes	-	no	yes	-	yes	yes	yes
**Every Pig**	-	no	-		no	-	yes	yes	yes
**Wash - disinfect**	yes	yes	-	yes	yes	yes	yes	yes	yes
**Wash - farrowing**	yes	yes	-	yes	yes	yes	yes	yes	yes
**Wash - gestation**	no	yes	-	no	no	no	no	no	no
**Wash - nursery/weaning**	yes	yes	-	yes	yes	yes	yes	yes	yes
**Wash - grower**	yes	no	-	no	yes	no	no	no	no
**Wash - finisher**	yes	no	-	no	yes	no	yes	yes	yes
**Downtime between batches**	yes	yes	-	no	yes	yes	yes	yes	yes
**Days of downtime**	2	4	-		4	2	7	7 to 10	7
**Same downtime each stage**	-	no	-	-	no	yes	yes	no	no

**Controlling pig flow**	-	-	-	-	clean truck requested for loading pigs on finishing sites	Transporter makes sure the pigs don't come back from the truck. The construction of the loading dock are so that there is very limited back flow.	2 people in farm and trucker in truck	Farrowing unit: truck to truck. One on farm truck that loads into another truck for transport; Nursery and finishing: narrow halls, multiple people	2 people in the farm and one on truck
**New equipment**	Disinfecting before entry	No rules	-	Empty room and disinfect afterwards	New if possible, no cleaning. Equipment for repairing gets a visual inspection without cleaning	Disinfection of all non-new equipment and tools if disinfection with liquid is possible	None really, but disinfection of equipment for maintenance	no	Disinfection and downtime of 24 hours before entering in barn
**Feed Biosecurity measures**	All delivered to one insulated location	Own guidelines	-	Drivers do not enter barn	feed in bags delivered to the farm, transfer to a boiler. Bulk feed delivered in an outdoor silo with automatic healer	no	no	no	no

**Other Biosecurity Measures**	-	-	-	Shower in and shower out, only semen is brought into the barn (no live animals)	Danish entries	Down time visitors - 36 hours; Truck for market pigs only comes on Monday and is visually inspected for hygiene. The bedding is only put in where the producer can see it.	Visitors must visit this barn before visiting other farms on the same day	-	none
Feed Type:									
**Feed Supplier**	Grand Valley, Wanstead (Corn)	Grand Valley	-	Floradale Feed Mill	Les nutriment Gillois inc.	Nutri-gene	Nutriment Gillois	Nutrition Athena	Alfred Couture
**Dry Sow 1**	mash	other (HMG)	-	pellet	mash	mash	mash	pellet	pellet
**Dry Sow 2**	mash	wet/dry	-	-	-	-	mash	-	pellet
**Lactation**	mash	-	-	pellet	mash	mash	mash, wet/dry	pellet	pellet
**Nursery 1**	pellet	pellet	-	pellet	pellet	mash	mash, wet/dry (in winter)	pellet	mash
**Nursery 2**	pellet	wet/dry	-	pellet	mash	mash	mash, wet/dry (in winter)	pellet	mash
**Nursery 3**	pellet	wet/dry	-	pellet	mash	mash	-	pellet	mash
**Nursery 4**		-	-	-	-	-	-	pellet	mash

**Grow-Finish 1**	pellet and liquid feed	other (HMG)	-	pellet	mash	mash	mash, wet/dry	wet/dry	pellets, wet/dry
**Grow-Finish 2**	pellet and liquid feed	other (HMG)	-	pellet	mash	mash	mash, wet/dry	wet/dry	pellets, wet/dry
**Grow-Finish 3**	pellet and liquid feed	other (HMG)	-	-	mash	-	mash, wet/dry	wet/dry	pellets, wet/dry
**Grow-Finish 4**	-	-	-	-	-	-	-	wet/dry	pellets, wet/dry
**Age Nursery 1 (weeks)**	-	-	-	4	3 to 5	3 to 4	4 to 7	3 to 4	4 to 5
**Weight Nursery 1 (kg)**	-	-	-	-	6.8	6 to 8	8 to 15	6 to 7.5	-
**Ingredients Nursery 1**	Ignite - Pellet	-	-	-	corn-soy	corn, soy, milk powder	corn-soy	corn, wheat, soya	corn, soy
**Age Nursery 2 (weeks)**	-	-	-	5	5 to 7	4 to 6	7 to 9	4 to 5.5	5 to 7
**Weight Nursery 2 (kg)**	-	-	-	-	8 to 12	8 to 12	15 to 23	7.5 to 12	-
**Ingredients Nursery 2**	Optimize - Pellet	-	-	-	corn-soy	corn, soy	corn-soy	corn, wheat, soy	corn, soy
**Age Nursery 3 (weeks)**	-	-	-	6	7 to 10	6 to 10	-	5.5 to 8	7 to 9
**Weight Nursery 3 (kg)**	-	-	-	-	12 to 25	12 to 25	-	12 to 20	-
**Ingredients Nursery 3**	Respond - Pellet	-	-	-	corn-soy	corn, soy, and wheat residue	-	corn, wheat, soya	corn, soy

**Age Nursery 4 (weeks)**	-	-	-	-	-	-	-	8 to 10	9 to 10
**Weight Nursery 4 (kg)**	-	-	-	-	-	-	-	20 to 28.5	25
**Ingredients Nursery 4**	-	-	-	-	-	-	-	corn, wheat, soya	corn, soy
**Age Grow-Finish 1 (weeks)**	-	-	-	8	10 to 14	-	-	10 to 13	-
**Weight Grow-Finish 1 (kg)**	180	-	-	-	25 to 50	-	-	28.5 to 45	25 oto 45
**Ingredients Grow-Finish 1**	Mash	-	-	-	corn-soy	corn, soy, and wheat residue	corn-soy	corn, wheat, soya	corn, soy
**Age Grow-Finish 2 (weeks)**		-	-	14	14 to 18	-	-	13 to 16	
**Weight Grow-Finish 2 (kg)**	32	-	-	-	50 to 75	-	-	45 to 65	45 to 65
**Ingredients Grow-Finish 2**	Mash	-	-	-	corn-soy	corn, soy, and wheat residue	corn-soy	corn, wheat, soya	corn, soy
**Age Grow-Finish 3 (weeks)**	-	-	-	-	18 to 22	-	-	16 to 21	
**Weight Grow-Finish 3 (kg)**	-	-	-	-	75 to 100	-	-	65 to 100	65 to 95
**Ingredients Grow-Finish 3**	-	-	-	-	corn-soy	corn, soy, and wheat residue	corn-soy	corn, wheat, soya	corn, soy

**Age Grow-Finish 4 (weeks)**	-	-	-	-	22 to 28	-	-	21 to 26	
**Weight Grow-Finish 4 (kg)**	-	-	-	-	100 to 135	-	-	100 to 125	95 to 130
**Ingredients Grow-Finish 4**	-	-	-	-	corn-soy	-	-	corn, wheat, soya	corn, soy
**Creep Feed**	yes	yes	-	yes	yes	yes	yes	yes	yes
**Creep Feed Introduction (days old)**	5	10	-	5	15	18	19	10	20
**Creep Feed Ingedients**	-	-	-	-	corn, soy	milk, corn, soy	-	-	corn and soy
<u>Feed Medication:</u>									
**Feed Medication**	yes	no	-	yes	no	no	no	yes	yes
**Type - Dry Sow**	-	-	-	-	-	-	-	Aureomycine 220	-
**Dose - Dry Sow**	-	-	-	-	-	-	-	3 kg/ton	-
**Type - Lactation**	Circo/Myco, Myco/Iron and Mly	-	-	Chlortetracycline	-	-	-	Aureomycine 220	Aivlosin
**Dose - Lactation**				220 mg/kg				4 kg/ton in first 2 weeks of lactation	1 kg/ton in week 1

**Type - Nursery 1**				Lincomycine 110				Aivlosin 17%	Sacox
**Dose - Nursery 1**				44 mg/kg				0.25 kg/ton in phases 1, 2, and 4	0.5 kg/ton in phase 3; 0.21 kg/ton in phase 4
**Type - Nursery 2**								Deracin 22%	Aureomycine 220
**Dose - Nursery 2**								2 kg/ton in phase 1, 2, and 4	2 kg/ton in phase 1 and 2
**Type - Grow/Finish 1**								Aivlosin 17%	Sacox
**Dose - Grow/Finish 1**								0.25 Kg/ton in phase 2	0.21 kg/ton for all phases
**Type - Grow/Finish 2**								Saccox 120	
**Dose - Grow/Finish 2**								0.21 kg/ton for all phases	
Water Medication:									
**Water Medication**	no	no	-	no	no	no	no	-	yes
**Type - Dry Sow**	-	-	-	-	-	-	-	-	-
**Dose - Dry Sow**	-	-	-	-	-	-	-	-	-

**Type - Lactation**	-	-	-	-	-	-	-	-	-
**Dose - Lactation**	-	-	-	-	-	-	-	-	-
**Type - Nursery**	-	-	-	-	-	-	-	-	Penicilline V
**Dose - Nursery**	-	-	-	-	-	-	-	-	20 mg/kg bodyweight in first week of nursery (week 4)
**Type - Grow/Finish**	-	-	-	-	-	-	-	-	-
**Dose - Grow/Finish**	-	-	-	-	-	-	-	-	-
**Vegetarian Diet**	no	yes	-	no	yes	yes	yes	yes	yes
**Blood Plasma Protein**	never	never	-	never	never	never	in the past	never	-
**Diet reformulation (y/n)**	-	-	-	no	no	yes	no	yes	no
**Vaccine 1:**									
**Name**	Circo/Myco Guard	Circo	-	Suvaxyn	Suvaxyn MH/HPS	Farrowsure B	Farrowsure B	Sequivity Influenza CérèsInfluenza	Ingelvac PRRS MLV
**Disease**	Circo/Myco	Circo	-	Mycoplasma	mycoplasm a and glasser	Parvo, lepto, erysipelas	PLE	-	PRRS

**Administration Method**		IM	-	IM	IM	IM	IM	IM	IM
**Administration Age**	-		-	3 weeks and booster at 6 weeks	gilts + sows, 4 weeks pre-farrow	all sows every four months (February, June, October)	5 weeks prefarrow	All sows in August once per year (gilts have 2 shots in quarantine)	entry of gilts, others in August and February
**Vaccine 2:**			-						
**Name**	FarrowSure Gold B	Myco	-	Ingelvac Circo Flex	Fostera PCV	Litterguard	Prosystem RCE	Farrowsure Gold	Circumvent PCV M G2
**Disease**	parvo, erysipelas, lepto	Myco	-	Circovirus	circovirus	E.coli, Clostridium perfingens	E. coli, Clostridium dificile, Rotavirus A	Parvo, lepto, erysipelia	Circovirus, mycoplasma
**Administration Method**	-	IM	-	IM	IM	IM	IM	IM	IM
**Administration Age**	-		-	3 weeks	gilt+sows 3 weeks pre-farrow	all sows 5 and 2 weeks before farrowing	5 and 2 weeks prefarrow for gilts and 2 weeks prefarrow sows	All sows in September once per year (gilts get 1 shot in quarantine)	gilts before insemination , others 3-4 weeks before farrowing
**Vaccine 3:**									
**Name**	-	Lawsonia	-	litterguard LTC	litterguard LTC	Respisure One	Circumvent PCV	ER bac plus	Farrowsure B
**Disease**	-	lawsonia	-	E. coli	ecoli, clostridium	Mycoplasma hyopneumoniae	Circovirus	Erysipelia	Parvovirus, Leptospirosis , Erysipelia

**Administration Method**	-	0	-	0		0	0	0	0
**Administration Age**	-	-	-	5 weeks and 2 weeks pre farrow	gilts+sows, 3 weeks pre farrow	second week in nursery	1 dose before first inseminatio n	6 weeks before farrowing	gilts 2 doses before insemination , others 6-8 weeks before farrowing
**Vaccine 4**		-							
**Name**	-	-	-	Farrow Sure Gold B	Ingelvac PRRS MLV	Circovac	Circumvent PCV	Porcine pillishield	Porcine pillishield
**Disease**	-	-	-	Parvovirus	PRRS	PCV 2	Circo virus	E. coli	E. coli
**Administration Method**	-	-	-	0	0	0	0	0	0
**Administration Age**	-	-	-	replacement gilts before breeding and weaned sows before breeding	females, twice/year	second week in nursery	21 days of age	5 weeks pre farrowing for gilts, 3 weeks pre farrowing for all sows	gilts 4 and 2 weeks before farrowing
**Vaccine 5**									
**Name**	-	-	-	-	Circovac	Enterisol ileitis	Coliprotec F4	Ingelvac PRRS MLV	Sequivity influenza, Flusure XP
**Disease**	-	-	-	-	circovirus	Lawsonia	E coli	PRRS	Influenza
**Administration Method**	-	-	-	-	0	2	2	0	0

**Administration Age**	-	-	-	-	3	sixth week of nursery	21 days of age	All sows in September once a year (gilts get 1 shot in quarantine)	Sequivity in March and August, Flusure 3 weeks later
**Administration Weight (kg)**	-	-	-	-	6	-	-	-	-
**Vaccine 6**	-	-	-	-	-	-	-	-	-
**Name**	-	-	-	-	Respisure one	-	-	Respisure one Porcillis Ileitis Circumvent PCV2	Circumvent PCV, Porcilis, Respisure-1
**Disease**	-	-	-	-	mycoplasm a	-	-	Mycoplasma hyopneumonia e, Lawsonia, Ileitis, circovirus	Circovirus, ileitis, mycoplasma
**Administration Method**	-	-	-	-	0	-	-	0	0
**Administration Age**	-	-	-	-	3	-	-	At weaning and 3 weeks later for all piglets	at weaning and 4 weeks later
**Administration Weight (kg)**	-	-	-	-	-	-	-	-	-
**Vaccine 7**									

**Name**	-	-	-	-	Enterisol ileitis	-	-	Ingelvac PRRS MLV	-
**Disease**	-	-	-	-	lawsonia	-	-	PRRS	-
**Administration Method**	-	-	-	-	2	-	-	0	-
**Administration Age**	-	-	-	-	10	-	-	3 weeks after weaning for all piglets	-
**Administration Weight (kg)**	-	-	-	-	40	-	-	-	-
**Vaccine 8**	-	-	-	-		-	-	-	-
**Name**	-	-	-	-	Ingelvac prrs MLV	-	-	-	-
**Disease**	-	-	-	-	PRRS	-	-	-	-
**Administration Method**	-	-	-	-	0	-	-	-	-
**Administration Age**	-	-	-	-	3	-	-	-	-
**PRRSV stage**	nursery	farrowing	-	-	Vaccine-like	gestation and farrowing	-	Farrowing and gestation	all
**PRRSV Last Test**	2019-04-30	10 years ago	-	-		29/04/2019	-	2017	29-9-2019
**Lawsonia stage**	-	nursery	-	-	finisher	finishing	-	-	-
**Lawsonia Last Test**	-	-	-	-	2017	not confirmed	-	-	-
**Swine Dysentry Stage**	-	-	-	-	0	-	-	-	-
**Swine Dysentry Last Test**	-	-	-	-		-	-	-	-
**Salmonella Stage**	-	-	-	-	0	-	-	-	-
**Salmonella Last Test**	-	-	-	-		-	-	-	-
**Coccidiosis Stage**	-	-	-	-	0	-	-	Suckling piglets	-

**Coccidiosis Last Test**	-	-	-	-		-	-	No tests	-
**E. coli Stage**	nursery	-	-	-	suckling piglets	suckling piglets	Nursery and finishing	-	-
**E. coli Last Test**	Jul-18	-	-	-	2017	2018-04-04	Jul-18	-	-
**Rotavirus Stage**	sows	-	-	-		suckling	-	nursery	suckling
**Rotavirus Last Test**	Jul-18	-	-	-		2018-04-04	-	Aug-16	31-03-2017
**Clostridium perfringens Stage**	-	-	-	-	sucking piglets	-	-	-	-
**Clostridium Perfringens Last Test**	-	-	-	-	2017	-	-	-	-
**Other Diseases**	-	myco circo	-	-	-	-	-	-	-
**Other Stage**	-	-	-	-	-	Influenza in all	Influenza and PCV	mycoplasma and influenza in sows	PCV2 in nursery
**Other Last Test**	-	-	-	-	-	not confirmed	Influenza in 2015, PCV 2 in 2016	2016	2018-03-05
**Outbreaks in last year**	yes	-	-	-	no	yes	yes	no	yes

**Outbreak comments**	PRRS 122-2 and have been dealing with RRRS 184 for some time. Fall of 2018 had myco break	-	-	-	-	PRRS (confirmed), E.coli (suspected), Influenza (suspected)	Lameness in suckling piglets	-	influenza in nursery, PRRS in nursery, rotavirus
**suspected confirmed Outbreaks**	no	-	-	-	-	-	no	-	Influenza and PRRS confirmed, Rotavirus not confirmed
