# Supplementary material for: Progression of swine fecal microbiota during early stages of life and its association with performance: a longitudinal study
Source: BMC Microbiol. 2024 May 25;24:182. doi: 10.1186/s12866-024-03336-y (PMC11127378; doi:10.1186/s12866-024-03336-y)
Supplement: Supplementary file 2 — Supplementary Material 2. [file 12866_2024_3336_MOESM2_ESM.pdf]

Additional file 2. Median (SD) relative abundances of **A)** the 17 phyla identified in piglet fecal swabs overall, **B)** families occurring with a minimum median relative abundance of 0.1%, and **C)** genera occurring with a minimum median relative abundance of 0.1%. Samples are grouped by system (conventional or raised without antibiotics), province (Ontario or Quebec), and sampling timepoint (TP1, TP2, TP3, or TP4) as identified by column headers. Differences between groups were assessed using Whitney-Mann test for system and province groupings, and Kruskal-Wallis test for sampling timepoint. Values are presented as Median (SD). Difference column denotes significant differences in relative abundance between groups corrected for multiple tests (* p < 0.05, ** p < 0.01, *** p < 0.001, **** p < 0.0001).

**2 A)**		System			Province			Sampling Timepoint				
Phylum	*Overall*	*Conventional*	*RWA*	*Difference*	*ON*	*QC*	*Difference*	*TP1*	*TP2*	*TP3*	*TP4*	*Difference*
Firmicutes	42.8 (12.5)	42.9 (12.5)	42.6 (12.5)	ns	42.2 (13.4)	43.4 (11.8)	ns	32.9 (10)	42.2 (11.7)	45.8 (11.8)	47 (10.9)	****
Bacteroidetes	33.5 (11.3)	33.6 (11.3)	33.4 (11.3)	ns	33.2 (11.5)	33.8 (11.2)	ns	32.1 (11.6)	31.9 (11.1)	29.3 (9.4)	40.9 (10.2)	****
Proteobacteria	9.2 (13)	8.9 (12.6)	9.6 (13.4)	ns	9.5 (12.5)	9.1 (13.3)	ns	15.2 (15.8)	10.3 (12.4)	8.5 (9.6)	4.2 (9.4)	****
Bacteria unclassified	1.2 (1.9)	1.2 (2.1)	1.2 (1.5)	ns	1.3 (2.1)	1.1 (1.6)	*	0.1 (0.9)	1.4 (2.1)	2.1 (2.2)	1.5 (1.2)	****
Fusobacteria	0.5 (7.2)	0.5 (6.9)	0.7 (7.4)	**	0.5 (7.8)	0.5 (6.6)	ns	11.2 (9.1)	0.7 (2.5)	0.3 (2.6)	0 (1)	****
Actinobacteria	0.4 (1.5)	0.4 (1.3)	0.5 (1.6)	***	0.5 (1.3)	0.4 (1.6)	ns	0.5 (0.9)	0.7 (1.4)	0.4 (2.3)	0.2 (0.6)	****
Synergistetes	0.2 (4)	0.2 (4.4)	0.1 (3.3)	***	0.2 (2.9)	0.1 (4.6)	ns	0 (0.5)	0.3 (3.5)	2.9 (6)	0.1 (0.8)	****
Spirochaetes	0.1 (3.2)	0.1 (3.6)	0.1 (2.6)	ns	0.2 (3.7)	0.1 (2.8)	***	0 (0.8)	0.1 (4)	0.3 (4.2)	0.8 (2.4)	****
Verrucomicrobia	0.1 (4.2)	0.1 (4)	0.1 (4.4)	ns	0.1 (4.4)	0.1 (4.1)	****	0 (1.5)	0.2 (6.8)	0.1 (3.9)	0.1 (1)	****
Planctomycetes	0 (0.5)	0 (0.6)	0 (0.4)	ns	0 (0.4)	0 (0.6)	ns	0 (0.2)	0 (0.5)	0.1 (0.7)	0.1 (0.4)	****
Lentisphaerae	0 (0.1)	0 (0.3)	0 (0.2)	ns	0 (0.1)	0 (0.2)	ns	0 (0)	0 (0)	0 (0.4)	0 (0.2)	****
Deferribacteres	0 (0.2)	0 (0.2)	0 (0.2)	*	0 (0.3)	0 (0.2)	ns	0 (0)	0 (0)	0 (0.1)	0 (0.4)	****
TM7	0 (0.5)	0 (0.2)	0 (0.1)	ns	0 (0.7)	0 (0.2)	*	0 (0)	0 (0)	0 (0.2)	0 (0.2)	****
Elusimicrobia	0 (0.2)	0 (0)	0 (0.1)	**	0 (0.2)	0 (0.1)	ns	0 (0)	0 (0)	0 (0.1)	0 (0.2)	****
Fibrobacteres	0 (0.1)	0 (0.2)	0 (0.1)	ns	0 (0.1)	0 (0.1)	ns	0 (0)	0 (0.2)	0 (0.2)	0 (0)	****

Tenericutes	0 (0.1)	0 (0.1)	0 (0)	ns	0 (0.1)	0 (0.1)	****	0 (0)	0 (0)	0 (0)	0 (0.2)	****
Chlamydiae	0 (0.2)	0 (0.6)	0 (0.1)	****	0 (0)	0 (0.3)	***	0 (0.9)	0 (0.2)	0 (0.1)	0 (0.1)	*

2 B)

Family	*Overall*	*Conventional*	*RWA*	*Difference*	*ON*	*QC*	*Difference*	*TP1*	*TP2*	*TP3*	*TP4*	*Difference*
Lachnospiraceae	10.6 (6.5)	**11 (6.4)**	**10.1 (6.7)**	ns	**9.6 (6.8)**	**11.1 (6.3)**	***	**8 (5)**	**11.7 (7.6)**	**11.7 (7)**	**11.2 (5)**	****
Bacteroidaceae	10.4 (10.9)	**10.9 (10.7)**	**9.9 (11.2)**	ns	**11.2 (10.4)**	**9.7 (11.3)**	ns	**21 (10.4)**	**15.6 (9.6)**	**10.9 (9.5)**	1.1 (3.2)	****
Ruminococcaceae	8.6 (6.2)	**8.9 (6.5)**	**8 (6)**	ns	**8.6 (6.2)**	**8.6 (6.3)**	ns	2.8 (4.7)	**8.9 (6.3)**	**9.4 (5.7)**	**11.8 (4.8)**	****
Prevotellaceae	6.7 (11.8)	**6.3 (12.1)**	**7.2 (11.5)**	ns	**6.3 (11.8)**	**7.1 (11.9)**	ns	3.3 (7.6)	4.2 (6.2)	4.3 (7)	**26.7 (10)**	****
Acidaminococcaceae	5.2 (4.5)	**5.3 (4.2)**	**5 (4.9)**	ns	**5.1 (4.3)**	**5.4 (4.7)**	ns	0.9 (2.7)	4.4 (4.5)	**6.8 (4.6)**	**7.2 (3.6)**	****
Porphyromonadaceae	4.6 (4.1)	5 (4.1)	4.3 (4.1)	*	4.7 (4)	4.6 (4.1)	ns	1.8 (4.9)	**5 (3.7)**	**4.7 (3.3)**	**6.4 (3.5)**	****
Enterobacteriaceae	4.2 (12.8)	4 (12.4)	4.6 (13.3)	ns	4.4 (12.3)	4.1 (13.3)	ns	**9.1 (16.6)**	**6.5 (12.6)**	4.4 (9.3)	0.5 (7.8)	****
Lactobacillaceae	2.2 (5.6)	2 (5.6)	2.5 (5.6)	ns	2 (5.8)	2.3 (5.4)	ns	2.8 (4.2)	2.6 (4.7)	1 (2.8)	2.6 (8.4)	****
Veillonellaceae	1.8 (5.3)	1.5 (5)	2.3 (5.5)	***	1.7 (5.4)	1.8 (5.2)	ns	**4.9 (5.7)**	0.9 (2.7)	1 (3.8)	2.3 (6.5)	****
Bacteria unclassified	1.2 (1.9)	1.2 (2.1)	1.2 (1.5)	ns	1.3 (2.1)	1.1 (1.6)	*	0.1 (0.9)	1.4 (2.1)	2.1 (2.2)	1.5 (1.2)	****
Clostridiales unclassified	1.1 (2.8)	1.2 (2.9)	1.1 (2.6)	ns	1.3 (3.3)	1.1 (2.3)	ns	0 (0.7)	1.1 (2.2)	2.6 (3.8)	2 (2.5)	****
Clostridiaceae 1	1.1 (3.2)	1.1 (3.1)	1.1 (3.4)	ns	1.1 (3.3)	1.1 (3.2)	ns	2.3 (4.7)	1.3 (2.9)	1.1 (2.5)	0.4 (1)	****
Desulfovibrionaceae	0.8 (0.8)	0.8 (0.8)	0.7 (0.7)	ns	0.7 (0.7)	0.8 (0.8)	ns	0.8 (0.8)	1.1 (0.8)	1.1 (0.6)	0.3 (0.4)	****
Bacteroidetes unclassified	0.6 (2)	0.7 (2.1)	0.6 (1.9)	ns	0.7 (2)	0.6 (2)	ns	0 (1)	0.5 (1.8)	1 (2)	1.9 (2.2)	****
Firmicutes unclassified	0.6 (2.3)	0.6 (2.4)	0.6 (2)	ns	0.6 (2.5)	0.7 (2)	ns	0 (0.5)	0.4 (1.4)	2.5 (2.9)	1.3 (2.2)	****
Fusobacteriaceae	0.5 (7.1)	0.4 (6.9)	0.7 (7.4)	*	0.5 (7.8)	0.5 (6.6)	ns	**11.2 (9.1)**	0.7 (2.5)	0.3 (2.6)	0 (1)	****
Streptococcaceae	0.4 (1.5)	0.4 (1.5)	0.5 (1.5)	ns	0.3 (1.4)	0.5 (1.6)	*	1.4 (1.8)	0.5 (1.7)	0.3 (0.9)	0.1 (0.6)	****
Pasteurellaceae	0.3 (1.8)	0.3 (1.5)	0.3 (2.2)	ns	0.3 (2.2)	0.2 (1.5)	ns	1.5 (3)	0.3 (1)	0.2 (0.6)	0 (0.8)	****
Coriobacteriaceae	0.2 (1.2)	0.2 (1.1)	0.2 (1.3)	ns	0.2 (1.1)	0.2 (1.3)	ns	0.1 (0.3)	0.3 (1.1)	0.2 (2)	0.2 (0.6)	****
Sutterellaceae	0.2 (0.8)	0.1 (0.8)	0.2 (0.7)	****	0.2 (0.8)	0.1 (0.7)	**	0.5 (1.2)	0.2 (0.6)	0.1 (0.3)	0.1 (0.3)	****
Rikenellaceae	0.2 (1.3)	0.2 (1.6)	0.2 (0.9)	ns	0.2 (1.2)	0.2 (1.4)	ns	0 (0.8)	0.7 (2)	0.6 (1)	0 (0.2)	****
Synergistaceae	0.2 (4)	0.2 (4.4)	0.1 (3.3)	***	0.2 (2.9)	0.1 (4.6)	ns	0 (0.5)	0.3 (3.5)	2.9 (6)	0.1 (0.8)	****

Erysipelotrichaceae	0.1 (0.2)	0.1 (0.3)	0.1 (0.2)	ns	0.1 (0.3)	0.1 (0.2)	ns	0.1 (0.2)	0.2 (0.3)	0.1 (0.2)	0.2 (0.3)	****
Bacteroidales unclassified	0.1 (1.6)	0.1 (1.1)	0.1 (2.1)	ns	0.1 (1.4)	0.1 (1.8)	ns	0 (1.7)	0.1 (1.5)	0.1 (2.1)	0.4 (1.3)	****
Eubacteriaceae	0.1 (1)	0.1 (1.2)	0.1 (0.7)	ns	0.1 (1.4)	0.1 (0.7)	**	0 (0.1)	0.1 (0.3)	0.1 (0.4)	0.5 (1.8)	****
Spirochaetaceae	0.1 (3.2)	0.1 (3.6)	0.1 (2.6)	ns	0.2 (3.7)	0.1 (2.8)	**	0 (0.8)	0.1 (4)	0.3 (4.2)	0.8 (2.4)	****
Campylobacteraceae	0.1 (1.9)	0.1 (1.7)	0.1 (2.1)	ns	0 (1.9)	0.1 (1.9)	***	0 (1)	0 (1.9)	0.1 (1.3)	0.4 (2.6)	****
Actinomycetaceae	0.1 (0.6)	0 (0.4)	0.1 (0.7)	ns	0.1 (0.5)	0 (0.7)	ns	0.1 (0.6)	0.2 (0.6)	0.1 (0.8)	0 (0)	****

2 C)

Genus	*Overall*	*Conventional*	*RWA*	*Difference*	*ON*	*QC*	*Difference*	*TP1*	*TP2*	*TP3*	*TP4*	*Difference*
Bacteroides	10.4 (10.9)	**10.9 (10.7)**	**9.9 (11.2)**	ns	**11.2 (10.4)**	**9.7 (11.3)**	ns	**21 (10.4)**	**15.6 (9.6)**	**10.9 (9.5)**	1.1 (3.2)	****
Ruminococcaceae unclassified	5.7 (5.5)	**5.9 (5.5)**	**5.5 (5.4)**	ns	**5.7 (5.5)**	**5.7 (5.5)**	ns	1 (4.1)	**7.5 (5.9)**	**8 (5.2)**	**6.2 (3.8)**	****
Prevotella	5.3 (9.5)	**4.7 (9.8)**	**6.2 (9.1)**	ns	**4.6 (9.2)**	**6 (9.7)**	ns	**2.8 (7.3)**	3.2 (5.4)	2.9 (5.4)	**19 (9.5)**	****
Phascolarctobacterium	4.8 (4.3)	**5 (4)**	**4.6 (4.7)**	ns	**4.8 (4)**	**4.9 (4.6)**	ns	0.9 (2.7)	**3.8 (4.2)**	**6.3 (4.5)**	**7.1 (3.4)**	****
Escherichia Shigella	4.1 (12.4)	**4 (12)**	**4.6 (12.9)**	ns	**4.2 (11.7)**	**4.1 (13)**	ns	**9 (16.2)**	**6.5 (12.2)**	**4.4 (8.9)**	0.4 (7.2)	****
Clostridium XlVa	2.7 (5.4)	3 (5.3)	2.3 (5.5)	ns	2.3 (4.9)	3 (5.7)	**	3 (4.3)	**5.9 (6.4)**	**4.7 (5.5)**	0.7 (1)	****
Lachnospiraceae unclassified	2.3 (4.9)	2.4 (4.6)	2.3 (5.3)	ns	2.3 (5.5)	2.4 (4.4)	ns	1.5 (1.8)	1.6 (6.4)	3.1 (6.4)	3.4 (2.7)	****
Lactobacillus	2.2 (5.6)	2 (5.6)	2.5 (5.6)	ns	2 (5.8)	2.3 (5.4)	ns	**2.8 (4.2)**	2.6 (4.7)	1 (2.8)	2.6 (8.4)	****
Bacteria unclassified	1.2 (1.9)	1.2 (2.1)	1.2 (1.5)	ns	1.3 (2.1)	1.1 (1.6)	*	0.1 (0.9)	1.4 (2.1)	2.1 (2.2)	1.5 (1.2)	****
Clostridiales unclassified	1.1 (2.8)	1.2 (2.9)	1.1 (2.6)	ns	1.3 (3.3)	1.1 (2.3)	ns	0 (0.7)	1.1 (2.2)	2.6 (3.8)	2 (2.5)	****
Clostridium sensu stricto	1.1 (3.2)	1 (3.1)	1.1 (3.4)	ns	1 (3.3)	1.1 (3.2)	ns	2.3 (4.7)	1.3 (2.9)	1 (2.5)	0.4 (1)	****
Prevotellaceae unclassified	0.8 (3.6)	0.8 (3.6)	0.9 (3.6)	ns	0.9 (3.8)	0.8 (3.4)	ns	0.1 (1.3)	0.4 (1.9)	0.8 (2.5)	**6.2 (3.7)**	****
Porphyromonadaceae unclassified	0.8 (2.4)	0.9 (2.2)	0.6 (2.6)	ns	0.8 (2.5)	0.7 (2.3)	ns	0.1 (1.5)	0.4 (1.2)	0.9 (1.8)	**3.9 (2.6)**	****
Desulfovibrio	0.7 (0.7)	0.7 (0.7)	0.6 (0.7)	ns	0.6 (0.7)	0.7 (0.7)	ns	0.7 (0.7)	0.9 (0.8)	0.9 (0.6)	0.3 (0.4)	****
Bacteroidetes unclassified	0.6 (2)	0.7 (2.1)	0.6 (1.9)	ns	0.7 (2)	0.6 (2)	ns	0 (1)	0.5 (1.8)	1 (2)	1.9 (2.2)	****
Firmicutes unclassified	0.6 (2.3)	0.6 (2.4)	0.6 (2)	ns	0.6 (2.5)	0.7 (2)	ns	0 (0.5)	0.4 (1.4)	2.5 (2.9)	1.3 (2.2)	****
Barnesiella	0.6 (2.4)	0.8 (2.4)	0.4 (2.4)	***	0.5 (2.1)	0.7 (2.6)	ns	0.1 (3.1)	0.5 (1.7)	0.6 (1.5)	1.7 (2.5)	****

Fusobacterium	0.5 (6.8)	0.4 (6.4)	0.6 (7.2)	*	0.5 (7.3)	0.5 (6.4)	ns	**10.8 (8.8)**	0.6 (2.4)	0.3 (2.5)	0 (0.8)	****
Butyricimonas	0.4 (1.5)	0.5 (1.5)	0.4 (1.4)	ns	0.5 (1.3)	0.4 (1.6)	ns	0.2 (1.7)	1.6 (1.6)	1 (1.2)	0 (0.1)	****
Streptococcus	0.4 (1.5)	0.4 (1.5)	0.5 (1.5)	ns	0.3 (1.4)	0.5 (1.6)	*	1.4 (1.8)	0.5 (1.7)	0.3 (0.9)	0.1 (0.6)	****
Lachnospiracea incertae sedis	0.3 (1.1)	0.3 (1.1)	0.4 (1.1)	ns	0.3 (1)	0.3 (1.1)	ns	0.5 (1.2)	0.1 (1.1)	0.1 (0.6)	1.2 (1)	****
Parabacteroides	0.3 (1.3)	0.3 (1.3)	0.3 (1.3)	ns	0.4 (1.4)	0.2 (1.2)	****	0.3 (0.7)	1 (1.7)	0.5 (1.4)	0 (0.1)	****
Oscillibacter	0.3 (0.7)	0.3 (0.7)	0.2 (0.6)	**	0.4 (0.7)	0.2 (0.7)	***	0 (0.3)	0.2 (0.6)	0.4 (0.7)	1 (0.7)	****
Butyricicoccus	0.3 (0.7)	0.3 (0.6)	0.3 (0.7)	ns	0.2 (0.6)	0.3 (0.7)	**	0.6 (1)	0.1 (0.3)	0.1 (0.3)	0.5 (0.6)	****
Veillonella	0.3 (3.8)	0.2 (3.6)	0.3 (4.1)	ns	0.3 (3.9)	0.2 (3.7)	ns	4.2 (5.7)	0.3 (1.6)	0.2 (0.6)	0 (1.1)	****
Clostridium XlVb	0.2 (0.7)	0.2 (0.7)	0.3 (0.6)	ns	0.3 (0.7)	0.2 (0.6)	ns	0.7 (0.8)	0.2 (0.6)	0.1 (0.3)	0.3 (0.5)	****
Alistipes	0.2 (1.3)	0.2 (1.6)	0.2 (0.9)	ns	0.2 (1.2)	0.2 (1.4)	ns	0 (0.8)	0.7 (2)	0.6 (1)	0 (0.2)	****
Sutterella	0.1 (0.7)	0.1 (0.8)	0.2 (0.7)	****	0.2 (0.8)	0.1 (0.7)	***	0.5 (1.2)	0.2 (0.6)	0.1 (0.3)	0.1 (0.1)	****
Dorea	0.1 (0.6)	0.2 (0.7)	0.1 (0.5)	***	0.2 (0.6)	0.1 (0.6)	ns	0 (0.1)	0.1 (0.4)	0.2 (0.7)	0.5 (0.8)	****
Blautia	0.1 (1.2)	0.2 (1.5)	0.1 (0.8)	****	0.1 (1.5)	0.1 (1.1)	ns	0 (0.2)	0.1 (0.4)	0.1 (0.5)	0.8 (2.1)	****
Bacteroidales unclassified	0.1 (1.6)	0.1 (1.1)	0.1 (2.1)	ns	0.1 (1.4)	0.1 (1.8)	ns	0 (1.7)	0.1 (1.5)	0.1 (2.1)	0.4 (1.3)	****
Eubacterium	0.1 (1)	0.1 (1.2)	0.1 (0.7)	ns	0.1 (1.4)	0.1 (0.7)	**	0 (0.1)	0.1 (0.3)	0.1 (0.4)	0.5 (1.8)	****
Actinobacillus	0.1 (1.2)	0.1 (0.8)	0.1 (1.6)	ns	0.1 (1.4)	0.1 (1)	**	0.6 (2.1)	0.1 (0.7)	0.1 (0.5)	0 (0.3)	****
Roseburia	0.1 (1.4)	0.1 (1.3)	0.2 (1.4)	****	0.1 (1.2)	0.1 (1.5)	ns	0 (1.3)	0 (0.7)	0.1 (0.6)	1.6 (1.7)	****
Flavonifractor	0.1 (0.2)	0.1 (0.3)	0.1 (0.2)	**	0.1 (0.2)	0.1 (0.3)	*	0.2 (0.4)	0 (0.2)	0.1 (0.2)	0.1 (0.1)	****
Collinsella	0.1 (0.7)	0.1 (0.7)	0.1 (0.8)	ns	0.1 (0.9)	0.1 (0.6)	****	0 (0.2)	0.1 (0.7)	0.1 (1.2)	0.2 (0.5)	****
Treponema	0.1 (3.2)	0.1 (3.6)	0.1 (2.6)	ns	0.1 (3.7)	0.1 (2.7)	**	0 (0.8)	0 (4)	0.2 (4.1)	0.8 (2.4)	****
Cloacibacillus	0.1 (3.2)	0.1 (3.5)	0 (2.9)	****	0.1 (2.6)	0.1 (3.7)	ns	0 (0.5)	0.3 (3.3)	1.8 (4.9)	0 (0.4)	****
Megasphaera	0.1 (2.8)	0.1 (2.6)	0.1 (3.1)	*	0 (2.7)	0.1 (2.9)	***	0 (0.9)	0.1 (1.3)	0.2 (2.5)	0.3 (4.6)	****
Clostridium IV	0.1 (0.2)	0.1 (0.3)	0.1 (0.2)	ns	0.1 (0.2)	0.1 (0.3)	ns	0 (0.1)	0.1 (0.2)	0 (0.1)	0.2 (0.3)	****
Pasteurella	0.1 (0.7)	0.1 (0.8)	0 (0.6)	ns	0 (0.8)	0.1 (0.7)	ns	0.3 (1)	0.1 (0.7)	0 (0.4)	0 (0.6)	****
Campylobacter	0.1 (1.9)	0.1 (1.7)	0.1 (2.1)	ns	0 (1.9)	0.1 (1.9)	***	0 (1)	0 (1.9)	0.1 (1.3)	0.4 (2.6)	****
Erysipelotrichaceae incertae sedis	0.1 (0.2)	0.1 (0.2)	0.1 (0.1)	ns	0.1 (0.2)	0.1 (0.2)	*	0 (0.1)	0.1 (0.3)	0 (0.1)	0.1 (0.1)	****
