# Supplementary material for: Progression of swine fecal microbiota during early stages of life and its association with performance: a longitudinal study
Source: BMC Microbiol. 2024 May 25;24:182. doi: 10.1186/s12866-024-03336-y (PMC11127378; doi:10.1186/s12866-024-03336-y)
Supplement: Supplementary file 3 — Supplementary Material 3. [file 12866_2024_3336_MOESM3_ESM.pdf]

Additional File 3:

i)

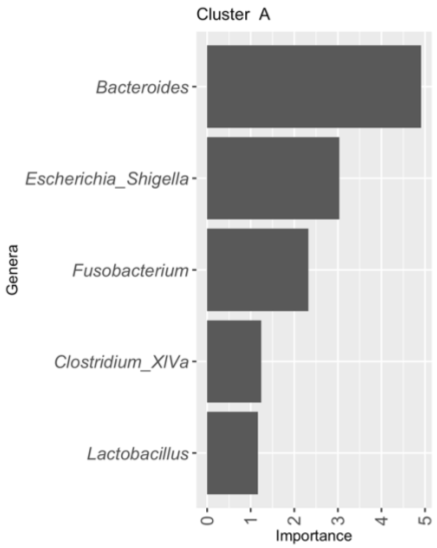

ii)

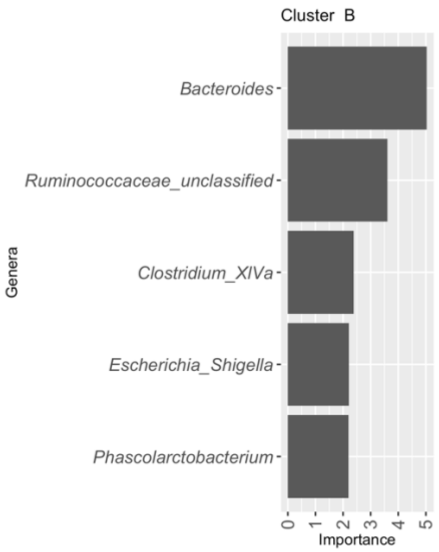

iii)

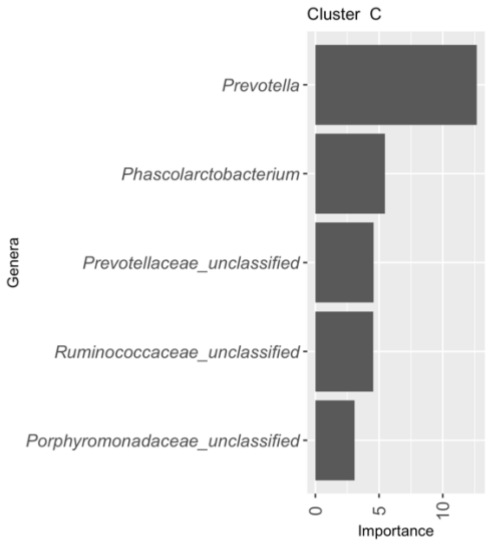

Figure S1. Dirichlet multinomial mixture model variable importance of top six genera driving differences between clusters. i) Cluster A community type, ii) Cluster B community type, and iii) Cluster C community type as identified by DMM where 93 % of samples followed the same pattern as hierarchical clustering.

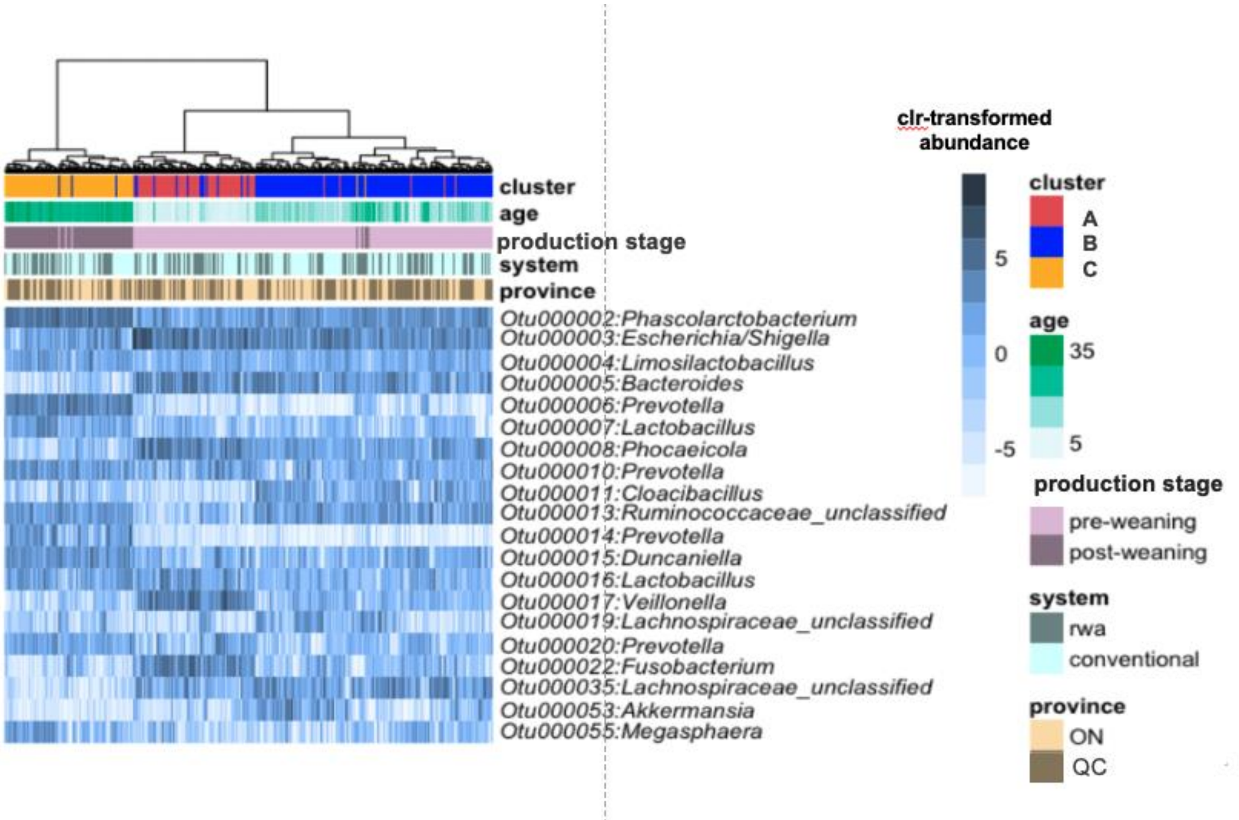

Figure S2. Dendrogram of hierarchical clustering using 263 core OTUs, and heatmap with OTU abundances. Each column represents a sample, top bars indicate stage assignment, piglet age and production stage (pre-weaning/post-weaning) at sample collection, and production system (conventional and raised without antibiotics) and province (Ontario/Quebec) information of each pig. Heatmap (below) displays the clr-transformed abundances of the top 20 most abundant OTUs within each stage.

Table S3. P-values of fixed effects and Akaike's Information Criteria (AIC) of model fit in univariate and multivariate regression models with body weight as the outcome variable. Multivariate models were built from significant fixed effects in univariate analysis.

**Fixed Effects**	**P-value**	**AIC value**
**Univariate Linear Regression**		
Age	< 0.0001	-105.5
TP1-TP2 path	< 0.0001	1076.5
TP1-TP3 path	< 0.0001	1094.1
System	0.78	1091.1
Province	< 0.0001	1074.8
Diarrhea occurrence (TP1/TP2/TP3)	0.15	1088.6
**Multivariate Linear Regression**	**P-value in order of model**	
(TP1-TP2 path) + Age	0.0050; < 0.0001	-101.2
(TP1-TP3 path) + Age	0.0164; < 0.0001	-79.9
Age + (TP1-TP2 path) + province	0.0026; < 0.0001; 0.0041	-103.4
Age + (TP1-TP3 path) + province	0.0099; < 0.0001; 0.0038	-82.4

Models were developed using lme() function in R, with sow as a random effect.
